# Estimates for sensory impairments requiring rehabilitation in China and globally: a comparative analysis of trends from 1990 to 2021 and future projections based on the GBD–WHO rehabilitation database 2021

**DOI:** 10.7189/jogh.16.04171

**Published:** 2026-07-10

**Authors:** Ding Chen, Zhenwei Qin, Zhongtai Wang, Tao Chen, Jingzhi Yang

**Affiliations:** 1 Gansu Province Hospital Rehabilitation Centre, Lanzhou, Gansu, China; 2 Lanzhou University Second Hospital, Lanzhou, Gansu, China

## Abstract

**Background:**

Sensory impairments, especially hearing and vision loss, are an increasing global public health concern. We examined the burden and trends of sensory impairment rehabilitation needs in China from 1990 to 2021 by age and sex, compared them with global data, and projected trends for the next 15 years.

**Methods:**

Using data from the World Health Organization rehabilitation need estimator, we analysed prevalence, years lived with disability (YLDs), and age-standardised rates (ASRs) at national and global levels. We calculated the average annual percent change (AAPC) using Joinpoint regression to identify significant trend shifts. We projected future trends using the Bayesian age-period-cohort model and decomposition analysis to assess the contributions of key drivers.

**Results:**

From 1990 to 2021, China experienced a more pronounced increase in the number of prevalent cases requiring sensory impairment rehabilitation (140.60%) than the global average (98.09%). The age-standardised prevalence rate (ASPR) increased significantly in China (AAPC = 0.2805), largely driven by hearing loss (AAPC = 0.4204), whereas the global ASPR increased only modestly (AAPC = 0.0797). Similarly, the age-standardised years lived with disability rate (ASYR) increased in China (AAPC = 0.1005) but declined globally (AAPC = –0.0955). Sensory impairments requiring rehabilitation were most prevalent among older adults (aged 55–79 years). Males were more affected at younger ages, whereas females were more affected at older ages. Decomposition analysis identified population ageing as the dominant driver in China. Projections indicate a slight decline in China’s ASPR over the next 15 years, accompanied by a decline in ASYR. Globally, both metrics are expected to decline slightly.

**Conclusions:**

Population ageing is the main driver of China’s rapidly growing need for sensory impairment rehabilitation, outpacing global trends. Urgent public health strategies are needed to strengthen rehabilitation systems and target older adults.

Sensory impairments, particularly hearing loss and vision loss, represent a significant and growing global public health challenge. As non-fatal health conditions, their primary impact is on functional ability, communication, social participation, and overall quality of life, contributing substantially to the global years lived with disability (YLDs) [1]. The Global Burden of Diseases (GBD), Injuries, and Risk Factors Study has been instrumental in quantifying this burden and has consistently ranked sensory disorders among the leading causes of disability worldwide [2,3].

The need for rehabilitation services, which are crucial for mitigating the disabling effects of sensory impairments, is escalating. This rise is largely driven by demographic and epidemiological transitions, most notably population ageing, as the prevalence of both hearing and vision loss increases markedly with age [4]. In response to this unmet need, the World Health Organization (WHO) has emphasised the integration of rehabilitation into health systems, a call underscored by the development of the GBD-WHO rehabilitation database. This database provides a standardised methodology for estimating the global scale of rehabilitation needs, enabling cross-country and temporal comparisons [5]. Recent studies utilising this framework have highlighted the substantial and increasing need for rehabilitation, with sensory impairments constituting a major component of this demand [6,7].

China, with the world’s largest population and one that is ageing at an unprecedented rate, faces a particularly acute challenge. Previous GBD-based analyses have documented a high and rising burden of sensory loss in China [8,9]. However, these studies mainly focused on the burden of disease in terms of prevalence and YLDs alone, without explicitly framing it within the critical context of rehabilitation needs. A comprehensive assessment that directly compares China’s trend of sensory impairment rehabilitation needs against the global backdrop is lacking. Such an analysis is essential to identify the specific drivers behind its national trends.

Furthermore, while the overall burden is recognised, a detailed, comparative analysis of long-term trends (1990–2021) and future projections, stratified by key demographic factors such as age and sex, remains insufficient. Understanding these temporal patterns and anticipating future scenarios are pivotal for evidence-based health system planning and resource allocation. Projection studies, particularly those employing robust Bayesian models, remain scarce in rehabilitation research but are critical for proactive policymaking [10].

Therefore, we aimed to systematically analyse and compare the temporal trends in China’s sensory impairment rehabilitation needs against global averages from 1990 to 2021, dissect the differential impacts of age and sex, identify key drivers like population ageing through decomposition analysis, and project China’s future trend over the next 15 years, thereby providing critical insights for shaping public health strategies and strengthening rehabilitation services.

## METHODS

### Data source

A comprehensive description of the methodology for the GBD-WHO rehabilitation database, including the underlying modelling strategies, has been previously reported [4]. The substantive data that formed the basis of this analysis adhered to the GRABDROP (Table S1 in the **Online Supplementary Document**), which includes recommendations for documenting data sources, estimation methods, statistical analyses, and statistical code [11]. In summary, the point prevalence and YLDs are estimated for 27 health conditions selected by a WHO Expert Panel on Rehabilitation [4]. The health conditions are grouped into eight GBD aggregate disease and injury categories: musculoskeletal disorders, neurological disorders, sensory impairments, mental disorders, chronic respiratory diseases, cardiovascular diseases, neoplasms, and COVID-19. The estimates for each condition are made for 204 countries and territories categorised into the six WHO regions: Africa, Eastern Mediterranean, European, South-East Asia, the Americas, and Western Pacific. The high-income countries (HICs) from each region were identified and grouped into a separate category according to World Bank criteria (Methods S1 in the **Online Supplementary Document**). Since this study utilises publicly available data, ethical approval or informed consent was not required.

In this secondary analysis, we obtained data on the prevalence of YLDs due to sensory impairments requiring rehabilitation in China and globally from 1990 to 2021 – stratified by age, sex, year, and health condition – using the WHO Rehabilitation Need Estimator. Sensory impairments included hearing loss and vision loss. Hearing impairment refers to the prevalence of hearing loss across a range of severities, as measured by the softest sound an individual can hear in their better ear – the average threshold across frequencies from 500 to 4000 Hz. Hearing loss severity is classified according to audiometric thresholds measured in decibels (dB). Hearing ability is categorised as follows: normal (0–19 dB), mild (20–34 dB), moderate (35–49 dB), moderately severe (50–64 dB), severe (65–79 dB), profound (80–94 dB), and complete (≥95 dB) [6]. The case definition for vision loss modelling is defined as visual acuity <6/18 (per the Snellen chart). Four severity levels of vision loss are modelled: blindness is defined as a visual acuity of <3/60 or a visual field of <10% around central fixation; severe vision loss corresponds to a visual acuity ≥3/60 and <6/60; moderate vision loss is characterised by a visual acuity ≥6/60 and <6/18; and near vision loss is defined as a near visual acuity of <6/12 (distance equivalent) [6].

### Derivation of prevalence and YLDs in the GBD-WHO study

In the GBD-WHO study, prevalence estimates for each condition were generated using the Bayesian meta-regression tool DisMod-MR, version 2.1 (WHO, Geneva, Switzerland), which analysed data from systematic reviews, health claims, surveys, registries, and surveillance systems, and ensured consistency across epidemiological parameters. YLDs, which measure the burden of non-fatal diseases and injuries, were calculated by multiplying the prevalence of each sequela by the estimated level of health loss, expressed as a disability weight ranging from ‘0’ (perfect health) to ‘1’ (death). Disability weights reflect disease severity and were derived from population surveys using pairwise comparisons of health states. Details on disability weight determination are available in prior publications [12-14]. We adjusted YLD estimates for comorbidity using simulation methods. We determined the 95% uncertainty interval (UI) for each estimate by the 2.5th and 97.5th percentiles from 1000 ordered draws.

### Joinpoint regression analysis

We used the Joinpoint Regression, version 4.9.1.0 (Statistical Methodology and Applications Branch, Surveillance Research Program, National Cancer Institute, Bethesda, Maryland, USA) to analyse temporal trends in the age-standardised prevalence rates (ASPR) and age-standardised YLDs rates (ASYR) for sensory impairments requiring rehabilitation in China and globally, and calculated the annual percent change (APC), average annual percent change (AAPC), and their corresponding 95% confidence intervals (CIs). The software fits a series of joined linear segments to the trend data on a logarithmic scale. In the analysis, we assumed a Poisson distribution for the rates to account for heteroscedasticity. We set the maximum number of joinpoints to five based on the number of data points (32 years) to avoid overfitting. We selected the optimal number of joinpoints and the best-fitting model using the Bayesian Information Criterion approach, as implemented in the software. We used the grid search method (the default algorithm) to locate the joinpoints. We assessed significance using a Monte Carlo permutation test with 4499 replicates, setting the significance level at *P* < 0.05. APC indicated segment-specific trends, while AAPC summarised the average for 1990–2021. We classified trends as declining (95% CI upper bound <0), increasing (lower bound >0), or stable. This method objectively identifies joinpoints while controlling for overfitting [15].

### Decomposition analysis

We employed the Das Gupta decomposition method to dissect the total changes in the number of prevalent cases and YLDs from 1990 to 2021 into contributions from three components – population growth, population ageing (changes in age structure), and epidemiological changes (changes in age-specific prevalence or YLD rates). We performed the analysis on count data only, not on age-standardised rates, as the latter are by design not influenced by population size or age structure. This approach enabled us to decompose the overall changes in burden into these key factors, thereby clarifying how demographic and epidemiological shifts have shaped trends over time. Unlike traditional methods, such as linear regression, which primarily focus on establishing relationships between variables, decomposition analysis enables a detailed assessment of each factor's independent contribution to overall changes in disease burden [16]. By breaking down these trends, we have obtained a more transparent picture of the underlying drivers of the global burden of rehabilitation needs for sensory impairments.

### Forecasting and projections

We projected future trends in rehabilitation needs for sensory impairments from 2022 to 2036 using a Bayesian age-period-cohort (BAPC) model, which was commonly used in GBD studies. This model combines historical trends with age-specific rates to predict future outcomes, accounting for temporal and demographic changes. To enhance accuracy and efficiency, we applied integrated nested Laplace approximations [17]. All projections include 95% UI, reflecting data variability, model assumptions, and inherent uncertainties, to provide a clear and comprehensive assessment of future rehabilitation needs.

### Statistical analysis

We performed data organisation using Microsoft Excel 2021 (Microsoft Corporation, Redmond, Washington, USA). We conducted trend analysis of ASRs using Joinpoint Regression, version 4.9.1.0 (Statistical Methodology and Applications Branch, Surveillance Research Program, National Cancer Institute, Bethesda, Maryland, USA) to calculate APC and AAPC [18,19], with a significance level of 0.05. We generated statistical analyses and graphical representations using *R*, version 4.4.0 (R Core Team, Vienna, Austria).

## RESULTS

### Rehabilitation needs for sensory impairments: prevalence in China and globally

In China, the number of prevalent cases requiring rehabilitation for sensory impairments increased from 68.77 million in 1990 to 165.46 million in 2021, representing a 140.60% increase. Globally, the number of cases rose from 372.27 million to 737.41 million, reflecting a 98.09% increase. The ASPR in China increased from 7755.67 per 100 000 persons to 8427.12, while globally, it rose from 8584.11 to 8777.10. From 1990 to 2021, the AAPC for the ASPR was 0.28 in China and 0.08 globally (**Table 1**).

**Table 1:** Estimated prevalence and YLDs for sensory impairments in need of rehabilitation in China and globally in 1990 and 2021

Conditions, location, and measure	1990, n (95% UI)		2021, n (95% UI)		1990–2021 AAPC, n (95% CI)
	**All-ages cases**	**ASR per 100 000**	**All-ages cases**	**ASR per 100 000**	
**Sensory impairments**					
China					
*Prevalence*	68 768 708 (63 064 142, 74 315 333)	7755.67 (7169.26, 8383.63)	165 457 550 (151 661 650, 179 412 948)	8427.12 (7790.86, 9070.76)	0.2805 (0.2426, 0.3184)
*YLDs*	5 567 940 (3 876 424, 7 709 750)	650.65 (453.89, 890.02)	12 799 485 (8 988 677, 17 671 866)	666.88 (464.75, 915.49)	0.1005 (0.0459, 0.1552)
Global					
*Prevalence*	372 265 325 (345 926 535, 397 958 664)	8584.11 (7990.37, 9140.89)	737 411 885 (683 609 549, 788 994 754)	8777.1 (8154.07, 9364.09)	0.0797 (0.0533, 0.1061)
*YLDs*	29 116 475 (20 193 516, 39 923 746)	692.78 (486.63, 942.81)	56 146 712 (38 945 114, 76 676 133)	670.96 (465.24, 913.53)	–0.0955 (–0.1277, –0.0634)
**Hearing loss**					
China					
*Prevalence*	46 709 025 (40 681 636, 52 425 290)	5349.18 (4743.94, 5952.53)	121 470 362 (107 415 353, 136 218 867)	6088.82 (5449.35, 6759.38)	0.4204 (0.3863, 0.4546)
*YLDs*	3 668 880 (2 526 929, 5 113 410)	421.2 (290.68, 583.9)	9 022 336 (6 159 841, 12 646 683)	468.78 (319.66, 655.01)	0.3492 (0.3217, 0.3768)
Global					
*Prevalence*	219 526 099 (194 415 690, 244 739 922)	5170.66 (4611.65, 5723.31)	466 751 067 (416 617 512, 518 124 627)	5554.32 (4962.09, 6145.08)	0.2376 (0.219, 0.2562)
*YLDs*	16 902 394 (11 503 229, 23 544 716)	398.88 (274.71, 554.2)	34 963 748 (23 913 935, 48 783 527)	419.15 (286.57, 585.32)	0.1623 (0.1552, 0.1695)
**Vision loss**					
China					
*Prevalence*	26 446 101 (24 277 938, 28 874 222)	3100.09 (2838.29, 3369.05)	59 163 151 (53 203 615, 65 693 178)	3098.55 (2831.89, 3384.79)	0.0411 (–0.0821, 0.1646)
*YLDs*	1 899 060 (1 348 974, 2 608 397)	229.45 (163.36, 313.8)	3 777 150 (2 662 962, 5 203 227)	198.11 (139.3, 270.18)	–0.3692 (–0.529, –0.2091)
Global					
*Prevalence*	176 92 1922 (163 479 427, 190 695 008)	4096 (3776.97, 4419.73)	329 049 586 (301 463 507, 358 558 428)	3918.7 (3600.64, 4252.78)	–0.1261 (–0.1533, –0.0989)
*YLDs*	12 214 081 (8 645 069, 16 717 100)	293.91 (208.86, 402.28)	21 182 964 (15 033 026, 29 039 803)	251.8 (178.65, 344.02)	–0.4846 (–0.5283, –0.4409)

AAPC – average annual percent change, ASR – age-standardised rate, CI – confidence interval, UI – uncertainty interval

For hearing loss, the number of prevalent cases requiring rehabilitation in China increased from approximately 46.7 million to 121.5 million, representing a 160.06% increase. Globally, cases rose from about 219.5 million to 466.8 million, an increase of 112.62%. The ASPR in China increased from 5349.18 to 6088.82 per 100 000 persons, while globally, it rose from 5170.66 to 5554.32 per 100 000 persons. The AAPC was 0.42 in China and 0.24 globally (**Table 1**).

For vision loss, the number of prevalent cases requiring rehabilitation in China increased from approximately 26.4 million to 59.2 million, representing a 123.71% increase. Globally, cases rose from about 176.9 million to 329.0 million, an 85.99% increase. The ASPR in China remained nearly stable, changing from 3100.09 to 3098.55 per 100 000 persons, whereas globally, it slightly declined from 4096.00 to 3918.7 per 100 000 persons. The AAPC was 0.04 in China, indicating a non-significant trend, and -0.13 globally, reflecting a statistically significant decline (**Table 1**).

### Rehabilitation needs for sensory impairments: YLDs in China and globally

YLDs attributable to sensory impairments requiring rehabilitation in China increased from 5.6 million to 12.8 million, representing a 129.88% increase. Globally, YLDs rose from 29.1 million to 56.1 million, a 92.83% increase. The ASYR in China increased from 650.65 to 666.88 per 100 000 persons, while globally, it decreased from 692.78 to 670.96. The AAPC for the ASYR was 0.10 in China, whereas –0.10 globally, indicating a statistically significant decrease (**Table 1**).

For hearing loss-related YLDs, China saw an increase from 3.7 million to 9.0 million, a 145.92% increase. Globally, YLDs rose from 16.9 million to 35.0 million, an increase of 106.86%. The ASYR in China increased from 421.20 to 468.78 per 100 000 persons, and globally, from 398.88 to 419.15 per 100 000 persons. The AAPC was 0.35 in China and 0.16 globally. For vision loss-related YLDs, China reported an increase from 1.9 million to 3.8 million, a 98.90% rise, while globally, YLDs increased from 12.2 million to 21.2 million, a 73.43% rise. The ASYR in China declined from 229.45 to 198.11 per 100 000 persons, and globally, from 293.91 to 251.80. The AAPC was –0.37 in China and –0.48 globally, both of which were statistically significant (**Table 1**).

### Joinpoint regression analysis of rehabilitation needs for sensory impairments in China and globally

**Figure 1:**
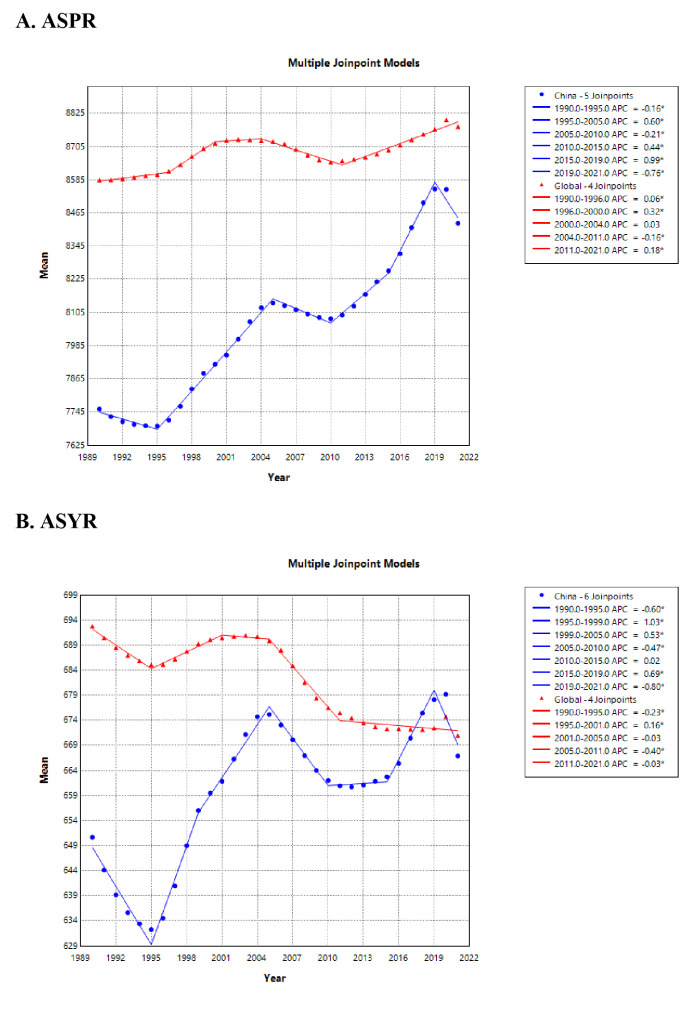
Annual percentage change in age-standardised rehabilitation needs for sensory impairments in China and globally from 1990 to 2021. **Panel A.** ASPR. **Panel B.** ASYR. ASPR – age-standardised prevalence rate, ASYR – age-standardised YLDs rate. *Indicates that the APC is significantly different from zero at the alpha at 0.05 level. The final selected model was six joinpoints.

In China, both the ASPR and ASYR demonstrated fluctuating yet overall upward trends over this period. Specifically, the ASPR initially declined from 1990 to 1995 (APC = –0.16), then rose significantly from 1995 to 2005 (APC = 0.60), followed by another decline from 2005 to 2010 (APC = –0.21), a renewed increase from 2010 to 2015 (APC = 0.44) and 2015 to 2019 (APC = 0.99), and a final marked decrease from 2019 to 2021 (APC = –0.76) (**Figure 1**, Panel A). A similar fluctuating pattern was observed for the ASYR, beginning with a decline from 1990 to 1995 (APC = –0.60), followed by increases from 1995 to 1999 (APC = 1.03) and 1999 to 2005 (APC = 0.53), a subsequent decline from 2005 to 2010 (APC = –0.47), a stable interval from 2010 to 2015 (APC = 0.02), a further rise from 2015 to 2019 (APC = 0.69), and a notable final downturn from 2019 to 2021 (APC = –0.80) (**Figure 1**, Panel B). Globally, the ASPR exhibited an overall increasing trend from 1990 to 2021, whereas the ASYR showed fluctuating but statistically significant declines.

In China, both rates displayed fluctuating but overall increasing trends. The ASPR showed an initial decline from 1990 to 2001 (APC = –0.03), followed by significant increases across multiple segments from 2001 to 2004 (APC = 1.58), 2004 to 2011 (APC = 0.20) and 2011 to 2019 (APC = 0.95), and a slight decrease at the end of the period from 2019 to 2021 (APC = –0.18). Similarly, the ASYR began with a decline from 1990 to 2000 (APC = –0.23), then rose through several phases from 2000 to 2005 (APC = 1.38), 2005 to 2011 (APC = 0.06), 2011 to 2015 (APC = 0.49), and 2015 to 2019 (APC = 1.18), before a final decline from 2019 to 2021 (APC = –0.37). Globally, both the ASPR and the ASYR for hearing loss rehabilitation needs showed overall upward trends from 1990 to 2021 (Figure S1 in the **Online Supplementary Document**).

In China, the ASPR for vision loss rehabilitation needs remained generally stable overall, whereas the ASYR exhibited a fluctuating but declining trend. The ASPR declined initially from 1990 to 1995 (APC = –0.57), rose markedly from 1995 to 2000 (APC = 2.33), then decreased again from 2000 to 2015 (APC = –0.52), increased briefly from 2015 to 2019 (APC = 1.03), and finally fell significantly from 2019 to 2021 (APC = –1.80). The ASYR also showed considerable fluctuation, beginning with a decline from 1990 to 1995 (APC = –1.18), followed by an increase from 1995 to 2000 (APC = 3.12), and subsequent sustained declines from 2000 to 2011 (APC = –1.21) and 2011 to 2021 (APC = –0.75). Globally, both ASPR and ASYR for vision loss rehabilitation needs demonstrated statistically significant decreasing trends with fluctuations between 1990 and 2021 (Figure S1 in the **Online Supplementary Document**).

### Rehabilitation needs for sensory impairments across different age groups in China and globally in 1990 and 2021

**Figure 2:**
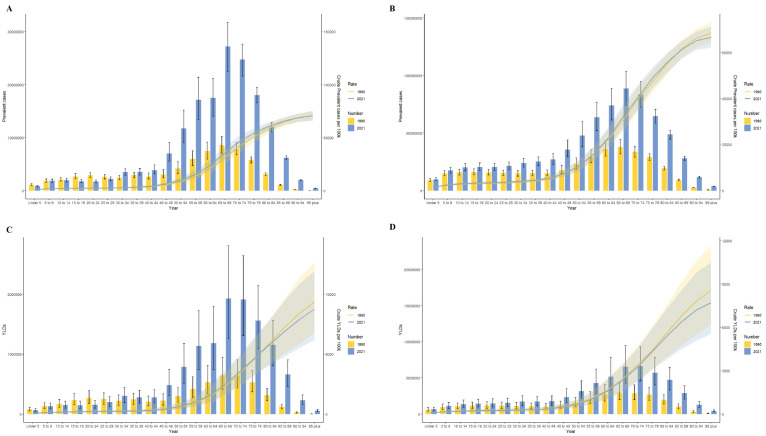
Comparison of the prevalence and YLDs counts, along with their crude rates, for sensory impairments rehabilitation needs by age group in China and globally from 1990 to 2021. **Panel A.** Prevalent cases and CPR in China. **Panel B.** Prevalent cases and CPR globally. **Panel C**. YLDs counts and CYR in China. **Panel D.** YLDs counts and CYR globally. CPR – crude prevalence rate, CYR – crude YLDs rate.

The crude prevalence rate (CPR) for sensory impairment rehabilitation needs increased monotonically with advancing age, with the highest rates observed in the oldest age groups. However, when examining the number of prevalent cases (counts), the peak burden was concentrated in the 55–79-year age range. This shift reflects the interaction between the age-specific rate and the population size in each age group; while the oldest-old (>80 years) have the highest rates, their smaller population size results in a lower total number of cases. A comparative assessment of CPR and CYR between China and the global population from 1990 to 2021 revealed broadly similar patterns, characterised by a modest increase among individuals under 45 years of age and a more pronounced rise in those aged 45 years and older (**Figure 2**, Panels A–D).

A similar age-dependent pattern was observed for hearing loss rehabilitation needs, with both CPR and CYR increasing progressively with age. The peak number of cases was consistently observed among adults aged 55–79 years. The decline in counts among the oldest-old (aged >90 years), may reflect a survival effect, where individuals with severe impairments have higher mortality, or it could be influenced by data sparsity in the oldest age groups within the GBD modelling framework. Longitudinal comparisons between China and global populations from 1990 to 2021 showed generally consistent trends – slight increases in CPR and CYR were seen in those aged <45 years, more substantial increases in the 45–90-year age group, and a decline among individuals aged >90 years.

For vision-loss rehabilitation needs, CPR and CYR also demonstrated a clear age-related increase in both China and globally. Again, the peak in case numbers occurred in the 55–79-year age group, which exhibited the greatest need for rehabilitation services. When comparing temporal trends between China and worldwide from 1990 to 2021, similar patterns emerged – modest increases in younger populations (aged <45 years) and more marked elevations in those aged ≥45 years (Figure S2 in the **Online Supplementary Document**).

### Gender differences in rehabilitation needs for sensory impairments across different age groups in China and globally

The prevalence and YLDs associated with rehabilitation needs for sensory impairments are stratified by sex and age group increased in 2021 compared with 1990, while overall trends remained consistent. The highest prevalence and YLDs counts were concentrated in the 65–74-year age group in China. Males predominated among individuals aged <65 years, whereas females accounted for a larger share among those aged ≥65 years. A similar sex-specific distribution was observed globally (Figure S3 in the **Online Supplementary Document**). Prevalence and YLD counts increased over this period in both sexes and in both China and globally. In China, males initially outnumbered females in the earlier years, but females gradually surpassed males in subsequent years. Globally, females consistently accounted for a higher burden throughout the period. The ASPR remained relatively stable in China but declined slightly globally, with males consistently exhibiting higher rates than females. Similarly, the ASYR decreased slightly in both China and globally, again with higher values among males (Figure S4 in the **Online Supplementary Document**).

For hearing loss, the prevalence and YLD counts of rehabilitation needs by sex and age group in 1990 and 2021, both measures increased in 2021 relative to 1990. Both in China and globally, the 65–74-year age group had the highest rehabilitation needs, males outnumbered females aged <70 years, whereas females predominated from age 70 onward. Longitudinal comparisons of hearing loss rehabilitation needs by sex from 1990 to 2021 revealed increasing trends in prevalence and YLD counts in both regions. In China, males consistently outnumbered females over time. Globally, males initially had higher rehabilitation needs, but females gradually surpassed them in later years. Both ASPR and ASYR showed slight increases over the study period in China and globally, with males consistently exhibiting higher rates (Figure S5 and S6 in the **Online Supplementary Document**).

Consistent with other sensory impairments, the 65–74-year age group bore the highest rehabilitation needs. In China, males predominated <55 years, while females accounted for a larger proportion among those aged ≥55 years. A similar sex distribution was seen globally. Temporal trends in vision loss rehabilitation needs by sex showed rising prevalence and YLD counts in both sexes and regions, with females consistently comprising the majority over time. In contrast to other impairments, both ASPR and ASYR for vision loss decreased in China and globally, with females consistently exhibiting higher standardised rates than males (Figures S7 and S8 in the **Online Supplementary Document**).

### The decomposition analysis of rehabilitation needs for sensory impairments in China and globally

A decomposition analysis was conducted to quantify the contributions of population ageing, population growth, and epidemiological changes to the temporal trends in the numbers of prevalent cases and YLDs of sensory impairment rehabilitation needs in China and globally from 1990 to 2021 (Figure S9 and Table S2 in the **Online Supplementary Document**). In China, population aging accounted for 67.45% of the increase in the number of prevalent cases and 72.76% of the increase in the number of YLDs, followed by population growth (22.07% for prevalent cases, 23.33% for YLDs), and epidemiological changes (10.48% for prevalent cases, 3.91% for YLDs), indicating that aging was the predominant driver of rising rehabilitation needs. Subgroup analyses by sex further revealed variations in the relative influence of these factors between males and females. Globally, the decomposition analysis similarly identified population aging and growth as the leading contributors to the increase in sensory impairment rehabilitation needs.

For hearing loss, population ageing contributed 64.89% to the increase in prevalent cases and 65.36% to that in YLDs in China, while population growth accounted for 20.31% and 21.50%, respectively. Epidemiological changes accounted for 14.80% of the change in prevalent cases and 13.14% of the change in YLDs. These results reflect the dominant role of ageing. Globally, the analysis likewise highlighted ageing and population growth as the principal factors underlying the increase in hearing loss-related rehabilitation needs (Figure S10 and Table S2 in the **Online supplementary Document**).

For vision loss, population ageing accounted for 75.56% of the increase in prevalent cases and 93.85% of the increase in YLDs in China, with population growth contributing 24.16% and 28.56%, respectively. Epidemiological changes had a minimal positive effect on prevalent cases (0.28%) and a negative contribution to YLDs (–22.41%), indicating that ageing was the overwhelming driver of rising needs. At the global level, population ageing and growth remained the key drivers of increased rehabilitation needs for vision loss during this period (Figure S10 and Table S2 in the **Online Supplementary Document**).

### Predicted trends in rehabilitation needs for sensory impairments in China and globally over the next fifteen years

In China, the overall ASPR (both genders combined) is projected to decrease slightly over the next 15 years, a trend consistent with declines in the ASPR for females and males. Consequently, the overall ASYR is projected to decrease, with similar declining trends observed in both females and males. All projections were generated using identical model parameters for comparability. Globally, slight decreases are predicted over the same period for both the overall ASPR and ASYR, with comparable reductions projected for females and males (Figure S11 and Tables S3 and S4 in the **Online Supplementary Document**).

In China, the overall ASPR is projected to remain stable, with a slight increase among females and a slight decrease among males. The overall ASYR is expected to decrease slightly, though females may experience a marginal increase while males show a slight decline. Globally, the overall ASPR and the ASPR for males are projected to remain stable, whereas the ASPR for females is projected to increase rapidly. The overall ASYR, along with the ASYR for females and males, is forecast to remain stable (Figure S12 and Table S3 in the **Online Supplementary Document**).

For vision loss, significant decreases are projected over the next 15 years in both the overall ASPR and ASYR, with consistent declines observed in both sexes in China. Globally, the overall ASPR is projected to decrease, with a decline among males but a rapid increase among females. The overall ASYR is forecast to decrease significantly, with similar reductions projected for both females and males (Figure S12 and Table S3 and S4 in the **Online Supplementary Document**).

## DISCUSSION

This comprehensive analysis reveals a substantial and growing burden of rehabilitation needs for sensory impairments in China and globally from 1990 to 2021. The rapid rise in cases in China, outpacing the global average, underscores an escalating public health challenge. Our decomposition analysis identified population ageing as the predominant driver of this increase, accounting for over two-thirds of the rise in both prevalent cases and YLDs in China. This finding aligns with global demographic transitions and is consistent with studies from other regions documenting the strong association between an ageing population and increased burden of sensory impairments [20].

A key finding of our study is the diverging trends between China and the global average. While the ASPR of sensory impairments requiring rehabilitation showed a significant increasing trend in China (AAPC = 0.2805), the global increase was more modest (AAPC = 0.0797). From 1990 to 2021, the AAPC for the ASPR of overall sensory impairments was 0.28% (95% CI = 0.24, 0.32) per year in China. While this annual percentage change is modest, it represents a cumulative relative increase of approximately 8.7% over the 32-year study period, translating to a substantial rise in the absolute number of people needing services. This contrasts with the global trend, where the AAPC of 0.08% per year reflects a much slower cumulative increase. This discrepancy may reflect China’s particularly rapid demographic transition, combined with its large population base. Similar patterns have been observed in other rapidly developing economies, where improved life expectancy has not been fully matched by the preservation of sensory health across the lifespan [8]. The Joinpoint regression analysis further illuminated this divergence, revealing more pronounced fluctuations in China’s ASPR and ASYR trends than in global patterns, potentially reflecting the impact of specific national health policies, environmental factors, or healthcare access issues unique to China’s development trajectory [21].

When examining specific sensory impairments, hearing loss requiring rehabilitation emerged as a particularly pressing concern in China, demonstrating both the highest increase in prevalent cases (160.06%) and the most rapid annual increase in ASPR (AAPC = 0.4204). This acceleration substantially exceeded the global trend (AAPC = 0.2376) and may be attributable to factors such as occupational noise exposure, environmental noise pollution, and low utilisation of hearing protection devices in China [22]. In contrast, vision loss showed more encouraging trends, with China’s ASPR remaining nearly stable and its ASYR declining significantly. The global decrease in vision loss ASPR (–0.1261%) suggests that worldwide public health interventions targeting preventable causes of vision impairment, such as cataract surgery programs and refractive error correction initiatives, may be yielding positive effects [23].

Our analysis reveals complex gender patterns that depend on whether one examines absolute burden (counts), crude rates, or age-standardised rates. We observed a consistent age-related increase in crude prevalence and YLD rates, with the peak number of cases occurring in the 55–79-year age group across all impairment types. This finding emphasises the critical importance of targeting rehabilitation services to older adult populations [24]. Gender distribution patterns differed notably by impairment type and age group. For overall sensory impairments and hearing loss specifically, males generally predominated in younger age groups, while females accounted for a larger share of the burden in older age groups. This is a classic example of the ‘female-male health-survival paradox,’ driven by two main factors: first, women have longer life expectancies and thus dominate the oldest age brackets where impairment rates are highest; second, the larger absolute number of women in these high-risk age groups inflates their contribution to total counts. Therefore, the higher standardised rates in men point to a need for understanding sex-specific risk factors, while the higher counts in older women highlight them as a key demographic group for targeted rehabilitation service delivery and resource allocation [25]. The consistently higher burden of vision loss among women in the oldest age groups warrants particular attention from health policymakers, especially given that women account for over half of the global blindness burden [26].

The decomposition analysis provided crucial insights into the underlying drivers of temporal trends. In China, population aging accounted for 67.45% and 72.76% of the increases in the number of prevalent cases and YLDs for sensory impairments requiring rehabilitation, respectively, substantially outweighing the contributions of population growth and epidemiological changes. This pattern was consistent across specific impairment types, with vision loss showing the most pronounced ageing effect (93.85% increase in YLDs). These findings highlight that demographic shift, rather than changes in age-specific risk, are the primary force behind the growing burden of sensory impairments [27]. This has profound implications for health system planning, suggesting that even with successful prevention efforts, rehabilitation service needs will continue to grow substantially due to inevitable demographic changes [28].

Our projections for the next 15 years suggest a mixed outlook. For sensory impairments requiring rehabilitation overall, China is projected to experience a decreasing ASPR and stable or declining sex-specific rates, a pattern that may reflect the complex interaction between demographic structure and epidemiological factors [29]. The projected rapid increase in hearing loss ASPR among females globally is concerning and merits further investigation into potential sex-specific risk factors [30]. The encouraging declines projected for vision loss ASPR and ASYR in most subgroups suggest that current prevention and treatment strategies may be effectively reducing the incidence or progression of vision impairments [31].

These findings have several important policy implications. First, the dominant role of population ageing in driving rehabilitation needs underscores the urgency of integrating sensory health services into geriatric care programs and developing age-friendly rehabilitation approaches. Second, the rapid rise in the hearing loss burden in China necessitates enhanced prevention strategies, including occupational noise-reduction policies in high-risk industries, alongside improved access to hearing aids through public financing or insurance subsidies. Third, the gender and age disparities identified call for targeted interventions for high-burden subgroups, particularly older women, which requires parallel investment in the rehabilitation workforce (audiologists, optometrists, low-vision therapists) and innovative financing mechanisms. The encouraging decline in age-standardised vision loss burden suggests that existing cataract and refractive programs have been effective; policy should now focus on expanding coverage to underserved rural populations and integrating low-vision rehabilitation services.

Future research should focus on elucidating the specific factors driving the rapid increase in hearing loss in China, developing cost-effective rehabilitation models for aging populations with sensory impairments, and exploring the effectiveness of targeted interventions for vulnerable subgroups. Additionally, an investigation into the barriers to accessing sensory rehabilitation services in different healthcare systems would help inform more equitable service delivery.

### Limitations

Several limitations of our study should be acknowledged. First, as with any analysis based on the GBD study, our results are subject to the inherent limitations of the input data, including potential gaps in data availability and quality for some regions and time periods; estimates may also vary across GBD cycles as new data become available or modelling strategies are refined. Second, our decomposition analysis could not account for all potential drivers of trends, such as changes in diagnostic practices or healthcare access. Third, although we used joinpoint regression to identify trends, we caution against interpreting these inflexion points as direct reflections of policy changes, given the ecological inference risks and model-based uncertainties inherent in the temporal GBD estimates. Fourth, all estimates are generated using DisMod-MR 2.1 rather than derived from direct measurements; therefore, observed temporal trends may reflect data artefacts or model recalibrations across GBD cycles rather than true underlying changes. Fifth, our findings may be sensitive to the choice of standard population and age groupings used in age-standardisation, although the GBD world standard population is widely accepted for international comparisons. Sixth, the projections assume that past trends will continue, which may not account for future disruptions or innovations in sensory impairment prevention and treatment; additionally, uncertainty compounds sequentially from the original GBD models through joinpoint analysis to the BAPC projections, meaning our confidence intervals may underestimate the full range of plausible future scenarios. Seventh, our estimates reflect the population that could benefit from rehabilitation – not actual service demand, which depends on eligibility, comorbidities, access to technology, and health system capacity.

## CONCLUSIONS

In conclusion, the escalating burden of sensory impairment rehabilitation needs, particularly in China, poses a formidable and distinct public health challenge, largely propelled by demographic ageing. The marked disparity between China’s rapid growth and the global average, coupled with the dominant role of population ageing as an explanatory factor, underscores the urgent need for proactive, integrated, and ageing-focused health policy planning. Future strategies must prioritise strengthening rehabilitation health systems, allocating resources efficiently, and implementing targeted interventions for high-burden groups such as the elderly to effectively address this growing need.

## Additional material


Online Supplementary Document
