# Supplementary material for: Estimates for sensory impairments requiring rehabilitation in China and globally: a comparative analysis of trends from 1990 to 2021 and future projections based on the GBD–WHO rehabilitation database 2021
Source: J Glob Health. 2026 Jul 10;16:04171. doi: 10.7189/jogh.16.04171 (PMC13348666; doi:10.7189/jogh.16.04171)
Supplement: Online Supplementary Document [file jogh-16-04171-s001.pdf]

Supplement to: Chen D, Qin Z, Wang Z, Chen T, Yang J. Estimates for sensory impairments requiring rehabilitation in China and globally: a comparative analysis of trends from 1990 to 2021 and future projections based on the GBD-WHO rehabilitation database 2021. J Glob Health. 2026;16:04171.

Supplementary Methods

Data Source

The Global Burden of Disease (GBD) 2021 provided a systematic and comprehensive annual assessment of prevalence, incidence, and years lived with disability (YLDs) for 371 diseases and injuries among 204 countries or territories from 1990 to 2021 (https://vizhub.healthdata.org/gbd-results/). The estimation process of GBD is based on identifying multiple relevant data sources, including censuses, household surveys, civil registration and vital statistics, disease registries, health service use, and other sources [1].

In our study, the worldwide and country-specific data on rehabilitation needs for sensory impairments were obtained from the GBD Rehabilitation Database, which was produced in collaboration with the World Health Organization (WHO). The data can be downloaded from the Global Health Data Exchange (GHDx) tool (https://vizhub.healthdata.org/rehabilitation/), and include prevalence, YLDs, and age-standardized prevalence and YLD rates from 1990 to 2021 by age, sex, and year.

Estimation of Rehabilitation Needs

In the latest GBD Rehabilitation Database, the global rehabilitation needs for a total of 27 disease causes, impairments, and bespoke aggregations of sequelae were estimated by providing the prevalence and YLDs.

The selection of the 27 conditions was based on the following procedures: (1) the conditions with the highest number of associated YLDs were identified; (2) the conditions for which rehabilitation was not essential and was usually indicated as a secondary intervention were excluded; (3) rehabilitation specialists discussed the above conditions and added any health conditions for which rehabilitation was a key intervention to the overall management plan. Finally, a total of 27 conditions were included and presented in 8 aggregate disease and injury categories, including musculoskeletal disorders, neurological disorders, sensory impairments, mental disorders, chronic respiratory diseases, cardiovascular diseases, neoplasms, and COVID-19. A detailed description of each health condition and its sequelae is provided in the study by Cieza et al. [2].

Annual numbers of prevalent cases, YLDs, and corresponding age-standardized rates (ASRs) (number per 100,000 persons) were used to describe rehabilitation needs for sensory impairments. A detailed description of the methodology for the GBD Rehabilitation Database, including the underlying modeling strategies, has been previously reported [2, 3]. ASRs were

computed using the world standard population developed for the GBD study, based on the following formula:

$$ASR = \sum_{i=1}^{n} \left(\frac{Ri \times P_{i}^{std}}{P_i} \right)$$

Where R_i is the disease incidence or mortality (i. e. age rate), P_i is the population in the studied population, P_i^{std} is the population proportion of the age group in the standard population, and n is the number of age group.

In summary, a Bayesian meta-regression modelling tool, DisMod-MR 2.1 (World Health Organization, Geneva, Switzerland), was used to estimate the prevalence of all conditions. This tool can evaluate all available data on the burden of a disease and enforce consistency between epidemiological parameters (details on pages 459–461, study by the GBD 2019 Diseases and Injuries Collaborators [3]). YLD, a measure of the burden of non-fatal diseases and injuries, was calculated by multiplying the prevalence of each sequela by the estimated level of health loss in the form of a disability weight that ranged from 0 (health) to 1 (death). Disability weight represents the severity of a disease and was identified from population surveys using a pair-wise comparison method between random pairs of health states. Uncertainty was propagated throughout this process by maintaining distributions of 1000 draws for each estimation stage. The 25th and 975th sorted values in the draw distributions were regarded as the lower and upper uncertainty intervals for mean estimates. Significant differences were established if 97.5% or more of the ordered 1000 values of the difference were on either side of zero.

Decomposition analysis

For the decomposition analysis, we used the decomposition method developed by Das Gupta to decompose the prevalence and YLDs for rehabilitation needs for sensory impairments in China and global caused by population ageing, population growth, and epidemiological changes [4]. The number of YLDs at each location was obtained from the following formula [5]:

$$YLD_{ay,\,py,\,ey} = \sum_{i=1}^{20} (a_{i,y} \times p_y \times e_{i.y})$$

Where $YLD_{ay,py,ey}$ represented YLDs based on the factors of age structure, population, and YLDs rate for specific year y; a_{iy} represents the proportion of population for the age category

i of the 20 age categories in given year y; p_y represents the total population in given year y; and $e_{i,y}$ represents YLDs rate given age category i in year y.

The contribution of each factor to the change in YLDs from 1990 to 2021 was defined by the effect of one factor changing while the other factors were held constant. For example, the effect of age structure was calculated as [5]:

$$[(YLD_{a2021, p1990, e1990} + YLD_{a2021, p2021, e2021})/3+ (YLD_{a2021, p1990, e2021} + YLD_{a2021, p2021, e1990})/6] - [(YLD_{a1990, p2021, e2021} + YLD_{a1990, p1990, e1990})/3+ (YLD_{a1990, p2021, e1990} + YLD_{a1990, p1990, e2021})/6]$$

Joinpoint regression analysis

The basic idea of the joinpoint regression model is to divide a long-term trend line into multiple segments through model fitting; each segment is defined by continuous linearity, and the intersections between distinct trend segments are known as turning points. Finally, we tested for significance using the Monte Carlo permutation method [6]. The joinpoint regression model can be expressed as a segmented function:

$$E[y \mid x] = e^{\beta_0 + \beta_1 x + \delta_1 (x - \tau_1)^+ + \ldots + \delta_k (x - \tau_k)^+}$$

Where y is the disease incidence or mortality rate. x indicates year. β_0 is the constant parameter. β_1 is the regression coefficient, k is the number of joinpoints. δ_k denotes the regression coefficient of the kth segment function.

The equation for the estimation of each segment's APC and the AAPC for that segment can be expressed as:

$$\ln(APC) = [\frac{y_{x+1} - y_x}{y_x}] * 100\% = (e^{\beta_1} - 1) * 100\%$$

$$\ln(AAPC) = (e^{\sum \omega_i \beta_i / \sum \omega_i} - 1) * 100\%$$

Projection of Rehabilitation Needs

Bayesian age-period-cohort analysis (BAPC) with integrated nested Laplace approximations (INLA) was used to project rehabilitation needs for sensory impairments in China and globally from 2022 to 2036. This method attributes separate effects to age, period, and cohort while extrapolating these effects to make projections. The APC model, first proposed by Mason in 1973 [7], is a common epidemiological tool that independently assesses the impact of age, period, and cohort effects. The age effect reflects developmental changes and

cumulative exposures associated with aging. The period effect represents temporal changes affecting all age groups simultaneously. The cohort effect captures differences among groups born in the same period. The APC model estimates individuals' age, birth cohort, and event period through the equation:

$$\log(\lambda_{ij}) = \mu + \alpha_i + \beta_j + \gamma_k$$

where μ, α_i, β_j, and γ_k represent the intercept, age effect, period effect, and cohort effect, respectively.

The cohort index k is derived from the age group index i, period index j, and the ratio of year spans between age groups and periods. In the Bayesian age-period-cohort framework, second-order random walks serve as smoothing priors for age, period, and cohort effects to project posterior rates. This modeling approach predicts each effect point through linear extrapolation from its two immediate predecessors, incorporating random variance from a normal distribution with zero mean. INLA facilitates BAPC and APC model implementation by approximating posterior marginal distributions directly, thereby avoiding Markov chain Monte Carlo (MCMC) sampling techniques and associated convergence issues [8].

References

1 Global incidence, prevalence, years lived with disability (YLDs), disability-adjusted life-years (DALYs), and healthy life expectancy (HALE) for 371 diseases and injuries in 204 countries and territories and 811 subnational locations, 1990-2021: a systematic analysis for the Global Burden of Disease Study 2021. *Lancet (London, England)* 2024, 403(10440):2133-2161.

2 Cieza A, Causey K, Kamenov K, Hanson SW, Chatterji S, Vos T: Global estimates of the need for rehabilitation based on the Global Burden of Disease study 2019: a systematic analysis for the Global Burden of Disease Study 2019. *Lancet (London, England)* 2021, 396(10267):2006-2017.

3 Global burden of 369 diseases and injuries in 204 countries and territories, 1990-2019: a systematic analysis for the Global Burden of Disease Study 2019. *Lancet (London, England)* 2020, 396(10258):1204-1222.

4 Liu K, Zhang K, Hu A, Li Y, Qin H, Sun W, Li X, Chen F, Liu T: Global burden of motor neuron disease: unraveling socioeconomic disparities, aging dynamics, and divergent future trajectories (1990-2040). *Journal of neurology* 2025, 272(6):390.

5 Xie Y, Bowe B, Mokdad AH, Xian H, Yan Y, Li T, Maddukuri G, Tsai CY, Floyd T, Al-Aly Z: Analysis of the Global Burden of Disease study highlights the global, regional, and national trends of chronic kidney disease epidemiology from 1990 to 2016. *Kidney international* 2018, 94(3):567-581.

6 Liu C, Zhu S, Zhang J, Wu P, Wang X, Du S, Wang E, Kang Y, Song K, Yu J: Global, regional, and national burden of liver cancer due to non-alcoholic steatohepatitis, 1990-2019: a decomposition and age-period-cohort analysis. *Journal of gastroenterology* 2023, 58(12):1222-1236.

7 Tian T, Zhu L, Fu Q, Tan S, Cao Y, Zhang D, Wang M, Zheng T, Gao L, Volontovich D *et al*: Needs for rehabilitation in China: Estimates based on the Global Burden of Disease Study 1990-2019. *Chinese medical journal* 2025, 138(1):49-59.

8 Riebler A, Held L: Projecting the future burden of cancer: Bayesian age-period-cohort analysis with integrated nested Laplace approximations. *Biometrical journal Biometrische Zeitschrift* 2017, 59(3):531-549.

S1. Outline of JoGH's Guidelines for Reporting Analyses of Big Data Repositories Open to the Public (GRABDROP) items

**JoGH guideline item**	**Purpose**
1. Please list all papers published by each co-author in previous three years that were based on secondary analysis of a big data repository<br><br>None of the authors of this article have published any papers based on secondary analyses of big data repositories in the past three years.	Preventing publications from possible 'paper mills' and authorships granted without genuine contribution
2. Please explain the key elements of your study design and the use of the available datasets that make your study an original scientific contribution<br><br>1. Original core study design<br>(1) China vs global direct comparative framework focusing specifically on sensory rehabilitation demand (not general sensory disease burden), stratified by metrics, disorder subtypes, age and sex<br>(2) Unique integrated four-method analytical pipeline: Joinpoint regression + Das Gupta decomposition + age-sex subgroup analysis + BAPC 15-year forecasting<br>(3) Specialized research perspective: only cases requiring rehabilitation (screened by WHO expert criteria) rather than all hearing/vision loss patients<br>(4) Unique timeline: 32-year historical data (1990–2021) plus 15-year long-term projection (2022–2036)<br>2. Original database application of GBD-WHO Rehabilitation Database<br>(1) Adopt exclusive rehabilitation screening standards unavailable in regular GBD studies<br>(2) Self-customized stratified decomposition & projection supplementary tables split by region, sex and disease<br>(3) Unified global standard population for fair China-global comparison; full uncertainty quantification across all analyses<br>(4) Data sourced from open-access WHO Rehabilitation Need Estimator for full reproducibility	Preventing duplicate publication where published information is reorganised and republished

3. Key original scientific findings & contributions<br>(1) First head-to-head comparison quantifying China’s much faster growth in sensory rehabilitation demand than global level<br>(2) Verified population ageing as the overwhelming driver of rising rehabilitation burden in China<br>(3) Uncovered differentiated age-sex burden distribution patterns for hearing and vision loss<br>(4) Provided 15-year forecasting data for sensory rehabilitation resource planning in China<br>(5) Built a reusable integrated statistical workflow template for national rehabilitation burden epidemiological research	
3. Please list all publications that addressed similar research questions in the same dataset and indicate where you cited them in your paper<br><br>1 Cieza A, Causey K, Kamenov K, Hanson SW, Chatterji S, Vos T. Global estimates of the need for rehabilitation based on the Global Burden of Disease study 2019: a systematic analysis for the Global Burden of Disease Study 2019. Lancet. 2021 Dec 19;396(10267):2006-2017.<br>[Ref 4]Cited: Background, Data Source (Methods), Discussion (global benchmark reference)<br>2 Tian T, Zhu L, Fu Q, Tan S, Cao Y, Zhang D, et al. Needs for rehabilitation in China: Estimates based on the Global Burden of Disease Study 1990-2019. Chin Med J (Engl). 2025 Jan 5;138(1):49-59.<br>[Ref 6]Cited: Background, Limitations (prior China-only short-term analysis contrast)<br>3 Zhang H, Chen Z, Liu S, Shi W, Cui X, Yuan W, et al. Trends of rehabilitation needs in 195 countries and regions from 1990 to 2021 and the impact of the COVID-19 pandemic on it: a systematic analysis of the global burden of disease study 2021. Int J Surg. 2025 Sep 10.<br>[Ref 10]Cited: Background, Discussion (cross-disease global trend comparison)<br>4 GBD 2019 Hearing Loss Collaborators. Hearing loss prevalence and years lived with disability, 1990-2019: findings from the Global Burden of Disease Study 2019. Lancet. 2021 Mar 13;397(10278):996-1009.<br>[Ref 30]Cited: Results, Discussion (hearing loss subgroup comparison)	Preventing duplicate publication where other groups have already conducted similar research in the same dataset

5 GBD 2019 Blindness and Vision Impairment Collaborators; Vision Loss Expert Group of the Global Burden of Disease Study. Trends in prevalence of blindness and distance and near vision impairment over 30 years: an analysis for the Global Burden of Disease Study. Lancet Glob Health. 2021 Feb;9(2):e130-e143.<br>[Ref 31]Cited: Results, Discussion (vision loss subgroup comparison)	
4. Please explain how you addressed multiple testing through an appropriately rigorous statistical threshold and indicate this in the methods section<br><br>We acknowledge that repeated subgroup analyses on the shared GBD-WHO database may generate overlapping weak findings seen in prior similar studies. We added strict multiple testing controls in Methods to avoid redundant positive results:<br>1. Joinpoint trend tests used 4499 Monte Carlo permutations to adjust the family-wise α=0.05 threshold, eliminating spurious transient trends.<br>2. All estimates required 95% uncertainty intervals entirely excluding zero as a second conservative filter, excluding borderline signals overreported in earlier same-dataset papers.This dual rigorous threshold reduces duplicate trivial significant outcomes from shared data.	Preventing publication of false positive associations which are of low value unless a rigorous statistical method accounted for multiple testing
Please declare to what extent have AI chatbots been used in developing your paper and to which parts of the paper did they contribute<br><br>AI chatbots merely assisted English language editing including grammar correction and sentence flow optimisation for all text sections. No AI tools participated in study design, data analysis, statistical inference, result interpretation or core scientific writing. All research work and substantive manuscript content were produced independently by authors; complete original data and analysis materials are available to ensure full transparency and reproducibility.	Preventing the improper use of AI chatbots in developing the key elements of the paper and ensuring reproducibility and transparency

Table S2 Prevalence and YLDs of sensory impairments in need of rehabilitation, with decomposition analysis categorized by China and Global

Condition	Location	Measure	Sex	Overll difference	Aging	Population	Epidemiological change
**Sensory impairments**	China	Prevalence	Both sexes	96688841.71	65220925.23 (67.45%)	21337172.5 (22.07%)	10130743.98 (10.48%)
		Prevalence	Females	49951579.79	34171921.84 (68.41%)	11200531.58 (22.42%)	4579126.375 (9.17%)
		Prevalence	Males	46737261.92	30931169.46 (66.18%)	10161396.38 (21.74%)	5644696.078 (12.08%)
		YLDs	Both sexes	7231545.24	5261738.529 (72.76%)	1687154.35 (23.33%)	282652.365 (3.91%)
		YLDs	Females	3799626.02	2814876.167 (74.08%)	894922.511 (23.55%)	89827.339 (2.36%)
		YLDs	Males	3431919.23	2437722.279 (71.03%)	794815.453 (23.16%)	199381.495 (5.81%)
	Global	Prevalence	Both sexes	365146560.1	143753310.2 (39.37%)	208619728.7 (57.13%)	12773521.22 (3.5%)
		Prevalence	Females	191614137.6	73095742.01 (38.15%)	109135057.6 (56.96%)	9383337.998 (4.9%)
		Prevalence	Males	173532422.5	70682666.06 (40.73%)	99580150.95 (57.38%)	3269605.493 (1.88%)
		YLDs	Both sexes	27030237.64	12171775.38 (45.03%)	16120591.98 (59.64%)	-1262129.711 (-4.67%)
		YLDs	Females	14394929.03	6241323.73 (43.36%)	8507603.335 (59.1%)	-353998.035 (-2.46%)
		YLDs	Males	12635308.61	5942623.225 (47.03%)	7623138.338 (60.33%)	-930452.951 (-7.36%)
**Hearing loss**	China	Prevalence	Both sexes	74761336.75	48516106.36 (64.89%)	15181729.67 (20.31%)	11063500.72 (14.8%)
		Prevalence	Females	37349030.24	24719193.33 (66.18%)	7696346.503 (20.61%)	4933490.406 (13.21%)
		Prevalence	Males	37412306.51	23732329.48 (63.43%)	7480672.061 (20%)	6199304.967 (16.57%)
		YLDs	Both sexes	5353455.76	3499128.164 (65.36%)	1150865.139 (21.5%)	703462.459 (13.14%)
		YLDs	Females	2686282.76	1800766.646 (67.04%)	585184.125 (21.78%)	300331.988 (11.18%)
		YLDs	Males	2667173	1698384.564 (63.68%)	565510.063 (21.2%)	403278.377 (15.12%)

Condition	Location	Measure	Sex	Overll difference	Aging	Population	Epidemiological change
	Global	Prevalence	Both sexes	247224968.5	94589577.59 (38.26%)	127908292.2 (51.74%)	24727098.71 (10%)
		Prevalence	Females	125727825.2	46768222.29 (37.2%)	64822469.11 (51.56%)	14137133.81 (11.24%)
		Prevalence	Males	121497143.3	48014102.02 (39.52%)	63109085.24 (51.94%)	10373956.02 (8.54%)
		YLDs	Both sexes	18061354.85	7061173.511 (39.1%)	9698674.795 (53.7%)	1301506.542 (7.21%)
		YLDs	Females	9278552.49	3532760.196 (38.07%)	4959893.094 (53.46%)	785899.202 (8.47%)
		YLDs	Males	8782802.36	3552539.532 (40.45%)	4741754.392 (53.99%)	488508.431 (5.56%)
**Vision loss**	China	Prevalence	Both sexes	32717049.96	24721297.29 (75.56%)	7904175.835 (24.16%)	91576.84 (0.28%)
		Prevalence	Females	18882120.67	14048912.98 (74.4%)	4562324.125 (24.16%)	270883.558 (1.43%)
		Prevalence	Males	13834929.29	10560888.47 (76.33%)	3383655.968 (24.46%)	-109615.148 (-0.79%)
		YLDs	Both sexes	1878089.48	1762610.365 (93.85%)	536289.211 (28.56%)	-420810.094 (-22.41%)
		YLDs	Females	1113343.26	1014109.522 (91.09%)	309738.387 (27.82%)	-210504.649 (-18.91%)
		YLDs	Males	764746.22	739337.715 (96.68%)	229305.39 (29.98%)	-203896.882 (-26.66%)
	Global	Prevalence	Both sexes	152127663.9	67159839.88 (44.15%)	95959511.09 (63.08%)	-10991687.06 (-7.23%)
		Prevalence	Females	85252414.9	35754164.86 (41.94%)	52906982.12 (62.06%)	-3408732.072 (-4%)
		Prevalence	Males	66875249.01	31230255.57 (46.7%)	43148000.57 (64.52%)	-7503007.122 (-11.22%)
		YLDs	Both sexes	8968882.79	5110601.864 (56.98%)	6421917.183 (71.6%)	-2563636.253 (-28.58%)
		YLDs	Females	5116376.54	2708563.534 (52.94%)	3547710.241 (69.34%)	-1139897.237 (-22.28%)
		YLDs	Males	3852506.26	2390083.693 (62.04%)	2881383.946 (74.79%)	-1418961.382 (-36.83%)

Table S2 Predicted ASPR and ASYR of sensory impairments in need of rehabilitation in China

Conditions	Years	ASPR(95% UI, per 100,000)			ASYR(95% UI, per 100,000)		
		Both sexes	Females	Males	Both sexes	Females	Males
**Sensory impairments**	2022	8396.04 (8250.04,8542.05)	8191.69 (8048.16,8335.21)	8600.4 (8451.92,8748.89)	672.01 (659.95,684.07)	655.84 (643.59,668.08)	680.43 (668.36,692.51)
	2023	8329.71 (8094.69,8564.73)	8104.98 (7867.94,8342.02)	8554.43 (8321.44,8787.43)	668.29 (649.48,687.1)	651.54 (632.29,670.79)	676.53 (657.51,695.55)
	2024	8263.56 (7916.1,8611.02)	8018.88 (7664.77,8372.99)	8508.24 (8167.43,8849.05)	664.55 (637.13,691.97)	647.24 (619.07,675.42)	672.6 (644.76,700.44)
	2025	8197.34 (7721.41,8673.26)	7933.09 (7446.14,8420.05)	8461.58 (7996.69,8926.47)	660.78 (623.44,698.12)	642.93 (604.51,681.35)	668.64 (630.66,706.62)
	2026	8131.34 (7514.36,8748.33)	7847.83 (7215.74,8479.92)	8414.86 (7812.98,9016.73)	657.02 (608.74,705.31)	638.62 (588.93,688.31)	664.7 (615.53,713.88)
	2027	8066.44 (7297.59,8835.28)	7763.85 (6976.21,8551.5)	8369.02 (7618.97,9119.07)	653.35 (593.24,713.47)	634.39 (572.54,696.24)	660.87 (599.57,722.16)
	2028	8002.22 (7072.3,8932.13)	7680.74 (6728.82,8632.66)	8323.7 (7415.79,9231.61)	649.72 (577,722.44)	630.2 (555.43,704.97)	657.08 (582.88,731.28)
	2029	7938.11 (6839.17,9037.04)	7597.9 (6474.34,8721.47)	8278.31 (7204,9352.62)	646.08 (560.08,732.07)	626 (537.64,714.36)	653.3 (565.5,741.11)

Conditions	Years	ASPR(95% UI, per 100,000)			ASYR(95% UI, per 100,000)		
		Both sexes	Females	Males	Both sexes	Females	Males
	2030	7873.84 (6598.89,9148.8)	7515.09 (6213.59,8816.59)	8232.6 (6984.19,9481.01)	642.41 (542.53,742.3)	621.77 (519.23,724.32)	649.52 (547.48,751.56)
	2031	7809.73 (6352.38,9267.07)	7432.59 (5947.58,8917.61)	8186.86 (6757.19,9616.53)	638.76 (524.42,753.09)	617.54 (500.27,734.81)	645.76 (528.9,762.62)
	2032	7746.57 (6100.81,9392.33)	7351.13 (5677.44,9024.81)	8142.01 (6524.17,9759.85)	635.18 (505.84,764.51)	613.36 (480.84,745.87)	642.1 (509.85,774.35)
	2033	7684.05 (5844.58,9523.51)	7270.29 (5403.63,9136.94)	8097.81 (6285.54,9910.07)	631.64 (486.81,776.46)	609.2 (460.97,757.42)	638.51 (490.36,786.66)
	2034	7621.64 (5583.91,9659.37)	7189.51 (5126.4,9252.62)	8053.77 (6041.42,10066.11)	628.11 (467.34,788.87)	605.03 (440.69,769.37)	634.97 (470.45,799.49)
	2035	7559.17 (5319.16,9799.17)	7108.64 (4846.23,9371.05)	8009.69 (5792.1,10227.29)	624.58 (447.47,801.68)	600.84 (420.02,781.67)	631.45 (450.13,812.77)
	2036	7496.99 (5050.96,9943.02)	7028.18 (4563.91,9492.45)	7965.8 (5538,10393.59)	621.06 (427.22,814.9)	596.64 (398.99,794.29)	627.98 (429.45,826.52)
**Hearing loss**	2022	6095.18 (5988.5,6201.86)	5727.69 (5633.49,5821.88)	6429.68 (6304.66,6554.71)	465.58 (458.31,472.85)	436.46 (429.56,443.36)	492.94 (484.41,501.47)

Conditions	Years	ASPR(95% UI, per 100,000)			ASYR(95% UI, per 100,000)		
		Both sexes	Females	Males	Both sexes	Females	Males
	2023	6099.61 (5939.96,6259.26)	5742.95 (5604.75,5881.15)	6423.6 (6231.32,6615.88)	465.17 (453.02,477.31)	437.06 (426.13,447.99)	491.56 (476.9,506.22)
	2024	6103.28 (5874.76,6331.79)	5757.63 (5561.69,5953.56)	6416.7 (6137.61,6695.79)	464.67 (446.36,482.99)	437.61 (421.51,453.72)	490.09 (467.75,512.43)
	2025	6106.04 (5796.89,6415.19)	5771.58 (5507.62,6035.54)	6408.82 (6028.63,6789.01)	464.08 (438.64,489.53)	438.09 (415.97,460.22)	488.53 (457.35,519.7)
	2026	6108.48 (5708.96,6508.01)	5785.24 (5444.69,6125.8)	6400.65 (5907.6,6893.7)	463.49 (430.11,496.87)	438.57 (409.71,467.44)	486.95 (445.98,527.93)
	2027	6111.01 (5612.46,6609.55)	5798.87 (5374.08,6223.67)	6392.66 (5776.39,7008.94)	462.93 (420.88,504.97)	439.06 (402.81,475.31)	485.42 (433.78,537.06)
	2028	6113.34 (5508.1,6718.57)	5812.22 (5296.37,6328.07)	6384.61 (5635.92,7133.3)	462.37 (411,513.73)	439.55 (395.34,483.75)	483.89 (420.82,546.96)
	2029	6114.87 (5396,6833.74)	5824.68 (5211.58,6437.79)	6375.91 (5486.52,7265.3)	461.73 (400.47,522.99)	439.97 (387.27,492.66)	482.3 (407.13,557.47)
	2030	6115.4 (5276.47,6954.33)	5836.04 (5119.9,6552.18)	6366.36 (5328.66,7404.07)	461.01 (389.31,532.7)	440.3 (378.63,501.98)	480.62 (392.72,568.51)

Conditions	Years	ASPR(95% UI, per 100,000)			ASYR(95% UI, per 100,000)		
		Both sexes	Females	Males	Both sexes	Females	Males
	2031	6115.41 (5150.28,7080.55)	5846.68 (5021.93,6671.43)	6356.5 (5163.25,7549.75)	460.27 (377.63,542.91)	440.61 (369.49,511.74)	478.94 (377.73,580.14)
	2032	6115.34 (5018.03,7212.65)	5856.86 (4918.07,6795.64)	6346.84 (4991.06,7702.62)	459.55 (365.47,553.63)	440.92 (359.88,521.95)	477.29 (362.22,592.36)
	2033	6115.07 (4880,7350.15)	5866.5 (4808.59,6924.4)	6337.27 (4812.43,7862.12)	458.84 (352.85,564.82)	441.2 (349.82,532.58)	475.67 (346.21,605.13)
	2034	6114.15 (4736.14,7492.17)	5875.07 (4693.33,7056.82)	6327.38 (4627.41,8027.34)	458.07 (339.76,576.39)	441.42 (339.3,543.54)	474.02 (329.69,618.34)
	2035	6112.37 (4586.53,7638.22)	5882.36 (4572.32,7192.39)	6316.96 (4436.18,8197.73)	457.23 (326.2,588.27)	441.54 (328.31,554.78)	472.31 (312.69,631.93)
	2036	6110.08 (4431.62,7788.55)	5888.69 (4445.99,7331.39)	6306.36 (4239.22,8373.49)	456.38 (312.24,600.53)	441.63 (316.91,566.35)	470.61 (295.26,645.96)
**Vision loss**	2022	3120.78 (3018.38,3223.19)	3312.19 (3193.65,3430.73)	2897.36 (2811.75,2982.98)	210.6 (201.26,219.94)	224.87 (214.4,235.34)	190.95 (182.78,199.12)
	2023	3055.5 (2895.06,3215.94)	3214.3 (3023.22,3405.38)	2866.38 (2740.08,2992.69)	209.13 (197.07,221.18)	222.5 (208.75,236.26)	189.77 (179.4,200.14)

Conditions	Years	ASPR(95% UI, per 100,000)			ASYR(95% UI, per 100,000)		
		Both sexes	Females	Males	Both sexes	Females	Males
	2024	2991.63 (2758.68,3224.59)	3119.35 (2839.88,3398.82)	2835.82 (2656.89,3014.75)	207.66 (191.99,223.34)	220.17 (202.03,238.3)	188.6 (175.32,201.89)
	2025	2929.12 (2615.27,3242.97)	3027.19 (2650.87,3403.52)	2805.63 (2566.23,3045.02)	206.22 (186.24,226.19)	217.85 (194.55,241.14)	187.46 (170.69,204.22)
	2026	2867.86 (2467.69,3268.04)	2937.72 (2459.47,3415.98)	2775.7 (2470.33,3081.06)	204.78 (179.99,229.57)	215.55 (186.52,244.57)	186.32 (165.66,206.99)
	2027	2808.06 (2317.6,3298.52)	2851.06 (2267.63,3434.5)	2746.26 (2370.64,3121.87)	203.36 (173.35,233.38)	213.28 (178.09,248.46)	185.21 (160.29,210.14)
	2028	2749.56 (2165.91,3333.2)	2767 (2076.5,3457.51)	2717.22 (2267.86,3166.58)	201.96 (166.38,237.54)	211.03 (169.35,252.71)	184.12 (154.65,213.59)
	2029	2692.29 (2013.31,3371.26)	2685.4 (1886.97,3483.83)	2688.56 (2162.49,3214.63)	200.57 (159.14,241.99)	208.8 (160.36,257.25)	183.04 (148.77,217.31)
	2030	2636.19 (1860.35,3412.04)	2606.15 (1699.76,3512.54)	2660.24 (2054.87,3265.62)	199.19 (151.67,246.71)	206.59 (151.15,262.04)	181.98 (142.69,221.27)
	2031	2581.2 (1707.44,3454.96)	2529.18 (1515.43,3542.92)	2632.17 (1945.19,3319.15)	197.82 (143.98,251.66)	204.4 (141.75,267.05)	180.94 (136.42,225.45)

Conditions	Years	ASPR(95% UI, per 100,000)			ASYR(95% UI, per 100,000)		
		Both sexes	Females	Males	Both sexes	Females	Males
	2032	2527.47 (1555.08,3499.86)	2454.58 (1334.51,3574.65)	2604.54 (1833.84,3375.24)	196.46 (136.1,256.83)	202.23 (132.21,272.26)	179.91 (129.99,229.84)
	2033	2474.87 (1403.57,3546.17)	2382.17 (1157.35,3606.99)	2577.28 (1721.02,3433.54)	195.12 (128.05,262.2)	200.08 (122.53,277.64)	178.91 (123.4,234.41)
	2034	2423.34 (1253.23,3593.45)	2311.81 (984.24,3639.39)	2550.37 (1606.96,3493.78)	193.8 (119.85,267.75)	197.96 (112.74,283.17)	177.92 (116.68,239.17)
	2035	2372.85 (1104.32,3641.38)	2243.45 (815.46,3671.44)	2523.8 (1491.83,3555.77)	192.49 (111.5,273.48)	195.85 (102.86,288.84)	176.97 (109.83,244.11)
	2036	2323.39 (957.05,3689.72)	2177.08 (651.22,3702.94)	2497.52 (1375.74,3619.29)	191.2 (103.02,279.37)	193.76 (92.9,294.63)	176.03 (102.85,249.21)

Table S3 Predicted ASPR and ASYR of sensory impairments in need of rehabilitation in China

Conditions	Years	ASPR(95% UI, per 100,000)			ASYR(95% UI, per 100,000)		
		Both sexes	Females	Males	Both sexes	Females	Males
**Sensory impairments**	2022	8396.04 (8250.04,8542.05)	8191.69 (8048.16,8335.21)	8600.4 (8451.92,8748.89)	672.01 (659.95,684.07)	655.84 (643.59,668.08)	680.43 (668.36,692.51)
	2023	8329.71 (8094.69,8564.73)	8104.98 (7867.94,8342.02)	8554.43 (8321.44,8787.43)	668.29 (649.48,687.1)	651.54 (632.29,670.79)	676.53 (657.51,695.55)
	2024	8263.56 (7916.1,8611.02)	8018.88 (7664.77,8372.99)	8508.24 (8167.43,8849.05)	664.55 (637.13,691.97)	647.24 (619.07,675.42)	672.6 (644.76,700.44)
	2025	8197.34 (7721.41,8673.26)	7933.09 (7446.14,8420.05)	8461.58 (7996.69,8926.47)	660.78 (623.44,698.12)	642.93 (604.51,681.35)	668.64 (630.66,706.62)
	2026	8131.34 (7514.36,8748.33)	7847.83 (7215.74,8479.92)	8414.86 (7812.98,9016.73)	657.02 (608.74,705.31)	638.62 (588.93,688.31)	664.7 (615.53,713.88)
	2027	8066.44 (7297.59,8835.28)	7763.85 (6976.21,8551.5)	8369.02 (7618.97,9119.07)	653.35 (593.24,713.47)	634.39 (572.54,696.24)	660.87 (599.57,722.16)
	2028	8002.22 (7072.3,8932.13)	7680.74 (6728.82,8632.66)	8323.7 (7415.79,9231.61)	649.72 (577,722.44)	630.2 (555.43,704.97)	657.08 (582.88,731.28)
	2029	7938.11 (6839.17,9037.04)	7597.9 (6474.34,8721.47)	8278.31 (7204,9352.62)	646.08 (560.08,732.07)	626 (537.64,714.36)	653.3 (565.5,741.11)
	2030	7873.84 (6598.89,9148.8)	7515.09 (6213.59,8816.59)	8232.6 (6984.19,9481.01)	642.41 (542.53,742.3)	621.77 (519.23,724.32)	649.52 (547.48,751.56)
	2031	7809.73 (6352.38,9267.07)	7432.59 (5947.58,8917.61)	8186.86 (6757.19,9616.53)	638.76 (524.42,753.09)	617.54 (500.27,734.81)	645.76 (528.9,762.62)
	2032	7746.57	7351.13	8142.01	635.18	613.36	642.1

Conditions	Years	ASPR(95% UI, per 100,000)			ASYR(95% UI, per 100,000)		
		Both sexes	Females	Males	Both sexes	Females	Males
		(6100.81,9392.33)	(5677.44,9024.81)	(6524.17,9759.85)	(505.84,764.51)	(480.84,745.87)	(509.85,774.35)
	2033	7684.05 (5844.58,9523.51)	7270.29 (5403.63,9136.94)	8097.81 (6285.54,9910.07)	631.64 (486.81,776.46)	609.2 (460.97,757.42)	638.51 (490.36,786.66)
	2034	7621.64 (5583.91,9659.37)	7189.51 (5126.4,9252.62)	8053.77 (6041.42,10066.11)	628.11 (467.34,788.87)	605.03 (440.69,769.37)	634.97 (470.45,799.49)
	2035	7559.17 (5319.16,9799.17)	7108.64 (4846.23,9371.05)	8009.69 (5792.1,10227.29)	624.58 (447.47,801.68)	600.84 (420.02,781.67)	631.45 (450.13,812.77)
	2036	7496.99 (5050.96,9943.02)	7028.18 (4563.91,9492.45)	7965.8 (5538,10393.59)	621.06 (427.22,814.9)	596.64 (398.99,794.29)	627.98 (429.45,826.52)
**Hearing loss**	2022	6095.18 (5988.5,6201.86)	5727.69 (5633.49,5821.88)	6429.68 (6304.66,6554.71)	465.58 (458.31,472.85)	436.46 (429.56,443.36)	492.94 (484.41,501.47)
	2023	6099.61 (5939.96,6259.26)	5742.95 (5604.75,5881.15)	6423.6 (6231.32,6615.88)	465.17 (453.02,477.31)	437.06 (426.13,447.99)	491.56 (476.9,506.22)
	2024	6103.28 (5874.76,6331.79)	5757.63 (5561.69,5953.56)	6416.7 (6137.61,6695.79)	464.67 (446.36,482.99)	437.61 (421.51,453.72)	490.09 (467.75,512.43)
	2025	6106.04 (5796.89,6415.19)	5771.58 (5507.62,6035.54)	6408.82 (6028.63,6789.01)	464.08 (438.64,489.53)	438.09 (415.97,460.22)	488.53 (457.35,519.7)
	2026	6108.48 (5708.96,6508.01)	5785.24 (5444.69,6125.8)	6400.65 (5907.6,6893.7)	463.49 (430.11,496.87)	438.57 (409.71,467.44)	486.95 (445.98,527.93)
	2027	6111.01 (5612.46,6609.55)	5798.87 (5374.08,6223.67)	6392.66 (5776.39,7008.94)	462.93 (420.88,504.97)	439.06 (402.81,475.31)	485.42 (433.78,537.06)
	2028	6113.34	5812.22	6384.61	462.37	439.55	483.89

Conditions	Years	ASPR(95% UI, per 100,000)			ASYR(95% UI, per 100,000)		
		Both sexes	Females	Males	Both sexes	Females	Males
		(5508.1,6718.57)	(5296.37,6328.07)	(5635.92,7133.3)	(411,513.73)	(395.34,483.75)	(420.82,546.96)
	2029	6114.87<br>(5396,6833.74)	5824.68<br>(5211.58,6437.79)	6375.91<br>(5486.52,7265.3)	461.73<br>(400.47,522.99)	439.97<br>(387.27,492.66)	482.3<br>(407.13,557.47)
	2030	6115.4<br>(5276.47,6954.33)	5836.04<br>(5119.9,6552.18)	6366.36<br>(5328.66,7404.07)	461.01<br>(389.31,532.7)	440.3<br>(378.63,501.98)	480.62<br>(392.72,568.51)
	2031	6115.41<br>(5150.28,7080.55)	5846.68<br>(5021.93,6671.43)	6356.5<br>(5163.25,7549.75)	460.27<br>(377.63,542.91)	440.61<br>(369.49,511.74)	478.94<br>(377.73,580.14)
	2032	6115.34<br>(5018.03,7212.65)	5856.86<br>(4918.07,6795.64)	6346.84<br>(4991.06,7702.62)	459.55<br>(365.47,553.63)	440.92<br>(359.88,521.95)	477.29<br>(362.22,592.36)
	2033	6115.07<br>(4880,7350.15)	5866.5<br>(4808.59,6924.4)	6337.27<br>(4812.43,7862.12)	458.84<br>(352.85,564.82)	441.2<br>(349.82,532.58)	475.67<br>(346.21,605.13)
	2034	6114.15<br>(4736.14,7492.17)	5875.07<br>(4693.33,7056.82)	6327.38<br>(4627.41,8027.34)	458.07<br>(339.76,576.39)	441.42<br>(339.3,543.54)	474.02<br>(329.69,618.34)
	2035	6112.37<br>(4586.53,7638.22)	5882.36<br>(4572.32,7192.39)	6316.96<br>(4436.18,8197.73)	457.23<br>(326.2,588.27)	441.54<br>(328.31,554.78)	472.31<br>(312.69,631.93)
	2036	6110.08<br>(4431.62,7788.55)	5888.69<br>(4445.99,7331.39)	6306.36<br>(4239.22,8373.49)	456.38<br>(312.24,600.53)	441.63<br>(316.91,566.35)	470.61<br>(295.26,645.96)
**Vision loss**	2022	3120.78<br>(3018.38,3223.19)	3312.19<br>(3193.65,3430.73)	2897.36<br>(2811.75,2982.98)	210.6<br>(201.26,219.94)	224.87<br>(214.4,235.34)	190.95<br>(182.78,199.12)
	2023	3055.5<br>(2895.06,3215.94)	3214.3<br>(3023.22,3405.38)	2866.38<br>(2740.08,2992.69)	209.13<br>(197.07,221.18)	222.5<br>(208.75,236.26)	189.77<br>(179.4,200.14)
	2024	2991.63	3119.35	2835.82	207.66	220.17	188.6

Conditions	Years	ASPR(95% UI, per 100,000)			ASYR(95% UI, per 100,000)		
		Both sexes	Females	Males	Both sexes	Females	Males
		(2758.68,3224.59)	(2839.88,3398.82)	(2656.89,3014.75)	(191.99,223.34)	(202.03,238.3)	(175.32,201.89)
	2025	2929.12 (2615.27,3242.97)	3027.19 (2650.87,3403.52)	2805.63 (2566.23,3045.02)	206.22 (186.24,226.19)	217.85 (194.55,241.14)	187.46 (170.69,204.22)
	2026	2867.86 (2467.69,3268.04)	2937.72 (2459.47,3415.98)	2775.7 (2470.33,3081.06)	204.78 (179.99,229.57)	215.55 (186.52,244.57)	186.32 (165.66,206.99)
	2027	2808.06 (2317.6,3298.52)	2851.06 (2267.63,3434.5)	2746.26 (2370.64,3121.87)	203.36 (173.35,233.38)	213.28 (178.09,248.46)	185.21 (160.29,210.14)
	2028	2749.56 (2165.91,3333.2)	2767 (2076.5,3457.51)	2717.22 (2267.86,3166.58)	201.96 (166.38,237.54)	211.03 (169.35,252.71)	184.12 (154.65,213.59)
	2029	2692.29 (2013.31,3371.26)	2685.4 (1886.97,3483.83)	2688.56 (2162.49,3214.63)	200.57 (159.14,241.99)	208.8 (160.36,257.25)	183.04 (148.77,217.31)
	2030	2636.19 (1860.35,3412.04)	2606.15 (1699.76,3512.54)	2660.24 (2054.87,3265.62)	199.19 (151.67,246.71)	206.59 (151.15,262.04)	181.98 (142.69,221.27)
	2031	2581.2 (1707.44,3454.96)	2529.18 (1515.43,3542.92)	2632.17 (1945.19,3319.15)	197.82 (143.98,251.66)	204.4 (141.75,267.05)	180.94 (136.42,225.45)
	2032	2527.47 (1555.08,3499.86)	2454.58 (1334.51,3574.65)	2604.54 (1833.84,3375.24)	196.46 (136.1,256.83)	202.23 (132.21,272.26)	179.91 (129.99,229.84)
	2033	2474.87 (1403.57,3546.17)	2382.17 (1157.35,3606.99)	2577.28 (1721.02,3433.54)	195.12 (128.05,262.2)	200.08 (122.53,277.64)	178.91 (123.4,234.41)
	2034	2423.34 (1253.23,3593.45)	2311.81 (984.24,3639.39)	2550.37 (1606.96,3493.78)	193.8 (119.85,267.75)	197.96 (112.74,283.17)	177.92 (116.68,239.17)
	2035	2372.85	2243.45	2523.8	192.49	195.85	176.97

Conditions	Years	ASPR(95% UI, per 100,000)			ASYR(95% UI, per 100,000)		
		Both sexes	Females	Males	Both sexes	Females	Males
		(1104.32,3641.38)	(815.46,3671.44)	(1491.83,3555.77)	(111.5,273.48)	(102.86,288.84)	(109.83,244.11)
	2036	2323.39<br>(957.05,3689.72)	2177.08<br>(651.22,3702.94)	2497.52<br>(1375.74,3619.29)	191.2<br>(103.02,279.37)	193.76<br>(92.9,294.63)	176.03<br>(102.85,249.21)

Table S4 Predicted ASPR and ASYR of sensory impairments in need of rehabilitation in Global

Conditions	Years	ASPR(95% UI, per 100,000)			ASYR(95% UI, per 100,000)		
		Both sexes	Females	Males	Both sexes	Females	Males
**Sensory impairments**	2022	8709.12 (4980.3,12437.93)	8651.99 (6640.18,10663.8)	8763.73 (5101.74,12425.71)	667.56 (661.56,673.56)	662.7 (656.67,668.73)	671.67 (665.3,678.03)
	2023	8680.83 (416.94,16944.71)	8627.93 (4283.93,12971.92)	8731.43 (593.58,16869.27)	665.6 (655.19,676.01)	661.09 (650.64,671.54)	669.29 (658.4,680.18)
	2024	8652.93 (-5074.29,22380.15)	8604.58 (1428.83,15780.34)	8699.36 (-4841.1,22239.82)	663.63 (647.71,679.55)	659.48 (643.51,675.46)	666.93 (650.37,683.49)
	2025	8620.76 (-11326.26,28567.78)	8580.72 (-1837.17,18998.62)	8662.23 (-11040.25,28364.7)	661.66 (639.42,683.9)	657.88 (635.56,680.19)	664.57 (641.5,687.63)
	2026	8594.66 (-18240.86,35430.18)	8557.75 (-5459.23,22574.73)	8632.63 (-17912.32,35177.59)	659.71 (630.45,688.97)	656.28 (626.92,685.64)	662.23 (631.95,692.5)
	2027	8560.77 (-25783.11,42904.65)	8531.92 (-9408.75,26472.58)	8595 (-25372.71,42562.71)	657.76 (620.86,694.66)	654.66 (617.62,691.7)	659.88 (621.76,698.01)
	2028	8533.58 (-33903.95,50971.11)	8508.76 (-13654.31,30671.84)	8564 (-33391.19,50519.19)	655.84 (610.74,700.93)	653.07 (607.79,698.36)	657.59 (611.06,704.12)
	2029	8506.87 (-42530.94,59544.69)	8486.46 (-18169.22,35142.14)	8533.34 (-41905.56,58972.23)	653.95 (600.16,707.73)	651.51 (597.48,705.54)	655.34 (599.9,710.78)
	2030	8475.42 (-51586.39,68537.23)	8463.23 (-22928.25,39854.7)	8497.68 (-50844.53,67839.89)	652.07 (589.14,715)	649.97 (586.73,713.21)	653.11 (588.31,717.91)
	2031	8450.6 (-61097.36,77998.56)	8441.39 (-27919.82,44802.6)	8469.64 (-60246.97,77186.25)	650.23 (577.72,722.74)	648.46 (575.57,721.36)	650.91 (576.31,725.52)
	2032	8418.7	8417.32	8434.17	648.41	646.96	648.73

Conditions	Years	ASPR(95% UI, per 100,000)			ASYR(95% UI, per 100,000)		
		Both sexes	Females	Males	Both sexes	Females	Males
		(-70991.6,87829)	(-33127.5,49962.14)	(-69999.62,86867.96)	(565.9,730.92)	(563.98,729.95)	(563.9,733.55)
	2033	8393.52 (-81337.42,98124.47)	8396.13 (-38554.25,55346.52)	8405.26 (-80180.31,96990.84)	646.66 (553.75,739.56)	645.52 (552.04,739.01)	646.62 (551.18,742.06)
	2034	8368.81 (-92059.37,108797)	8375.63 (-44180.92,60932.18)	8376.72 (-90722.19,107475.64)	644.95 (541.28,748.62)	644.13 (539.78,748.49)	644.58 (538.15,751)
	2035	8339.5 (-103069.42,119748.42)	8353.89 (-49985.74,66693.51)	8343.88 (-101548.13,118235.89)	643.27 (528.49,758.05)	642.77 (527.18,758.35)	642.58 (524.83,760.32)
	2036	8316.27 (-114470.82,131103.36)	8333.75 (-55975.05,72642.56)	8317.67 (-112768.92,129404.27)	641.64 (515.42,767.86)	641.44 (514.28,768.61)	640.61 (511.21,770.01)
**Hearing loss**	2022	5517.65 (5367.59,5667.71)	5494.99 (5441.58,5548.41)	5788.76 (5734.14,5843.38)	417.38 (412.93,421.82)	400.64 (396.38,404.89)	435.25 (430.41,440.08)
	2023	5514.11 (5192.33,5835.9)	5792.08 (5699.85,5884.3)	5789.48 (5697.84,5881.12)	417.44 (410.11,424.78)	401.12 (394.14,408.11)	434.9 (426.9,442.89)
	2024	5509.63 (4978.95,6040.31)	6206.26 (6059.54,6352.97)	5789.91 (5651.53,5928.29)	417.46 (406.47,428.46)	401.57 (391.13,412.02)	434.49 (422.49,446.49)
	2025	5502.49 (4732.73,6272.25)	6743.89 (6524.96,6962.81)	5789.89 (5597.45,5982.32)	417.44 (402.2,432.67)	401.99 (387.53,416.45)	434.03 (417.41,450.65)
	2026	5498.82 (4462.99,6534.65)	7416.19 (7102.99,7729.39)	5789.97 (5537.22,6042.72)	417.43 (397.46,437.39)	402.42 (383.47,421.38)	433.59 (411.82,455.36)
	2027	5491.87 (4164.21,6819.53)	8239.03 (7803.33,8674.73)	5790.2 (5471.4,6109)	417.42 (392.28,442.57)	402.85 (378.98,426.73)	433.17 (405.78,460.57)
	2028	5487.98	9230.28	5790.71	417.42	403.28	432.75

Conditions	Years	ASPR(95% UI, per 100,000)			ASYR(95% UI, per 100,000)		
		Both sexes	Females	Males	Both sexes	Females	Males
		(3844.7,7131.25)	(8636.44,9824.12)	(5400.74,6180.68)	(386.7,448.14)	(374.1,432.47)	(399.31,466.19)
	2029	5483.88<br>(3504.07,7463.69)	10409.96<br>(9613.1,11206.82)	5791.2<br>(5325.39,6257.01)	417.39<br>(380.74,454.05)	403.7<br>(368.85,438.55)	432.29<br>(392.42,472.17)
	2030	5478.21<br>(3143.34,7813.09)	11800.97<br>(10744.83,12857.11)	5791.47<br>(5245.49,6337.45)	417.33<br>(374.4,460.25)	404.09<br>(363.25,444.93)	431.8<br>(385.14,478.45)
	2031	5474.32<br>(2765.86,8182.78)	13430.59<br>(12044.86,14816.32)	5791.74<br>(5161.4,6422.08)	417.27<br>(367.75,466.8)	404.49<br>(357.33,451.65)	431.32<br>(377.54,485.1)
	2032	5467.06<br>(2368.09,8566.02)	15332.38<br>(13529.27,17135.48)	5792.12<br>(5073.31,6510.93)	417.23<br>(360.79,473.66)	404.88<br>(351.09,458.67)	430.85<br>(369.62,492.09)
	2033	5463.13<br>(1954.77,8971.48)	17542.09<br>(15213.57,19870.61)	5792.89<br>(4981.71,6604.07)	417.19<br>(353.54,480.84)	405.29<br>(344.56,466.01)	430.4<br>(361.4,499.4)
	2034	5459.53<br>(1525.49,9393.56)	20098.17<br>(17112.24,23084.1)	5793.91<br>(4886.68,6701.14)	417.15<br>(346,488.29)	405.69<br>(337.75,473.63)	429.94<br>(352.88,506.99)
	2035	5455.6<br>(1081.07,9830.14)	23044.07<br>(19240.23,26847.91)	5794.96<br>(4788.18,6801.74)	417.08<br>(338.18,495.99)	406.08<br>(330.66,481.5)	429.45<br>(344.07,514.83)
	2036	5451.74<br>(622.42,10281.06)	26432.3<br>(21615.45,31249.15)	5795.95<br>(4686.21,6905.7)	417.03<br>(330.1,503.95)	406.47<br>(323.3,489.64)	428.98<br>(335.01,522.95)
**Vision loss**	2022	3883.68<br>(3761.59,4005.76)	4840.78<br>(4792.72,4888.83)	3685.46<br>(3646.34,3724.59)	250.97<br>(248.32,253.62)	262.78<br>(259.92,265.64)	237.43<br>(234.7,240.16)
	2023	3856.95<br>(3593.43,4120.47)	6200.31<br>(6103.49,6297.13)	3671.9<br>(3607.08,3736.71)	249.24<br>(244.77,253.71)	261<br>(256.17,265.84)	235.73<br>(231.3,240.15)
	2024	3830.86	8915.42	3659.04	247.58	259.29	234.1

Conditions	Years	ASPR(95% UI, per 100,000)			ASYR(95% UI, per 100,000)		
		Both sexes	Females	Males	Both sexes	Females	Males
		(3396.5,4265.21)	(8727.85,9102.99)	(3561.99,3756.08)	(240.85,254.31)	(251.99,266.59)	(227.55,240.66)
	2025	3805.25 (3176.21,4434.29)	14852.08 (14462.7,15241.45)	3646.59 (3512.52,3780.67)	245.99 (236.67,255.31)	257.64 (247.53,267.75)	232.56 (223.57,241.55)
	2026	3779.68 (2935.74,4623.63)	30386.63 (29397.55,31375.7)	3633.63 (3458.48,3808.78)	244.37 (232.2,256.54)	255.96 (242.75,269.17)	230.99 (219.31,242.66)
	2027	3753.82 (2677.33,4830.31)	80595.65 (77306.98,83884.32)	3620.2 (3400.31,3840.09)	242.73 (227.48,257.99)	254.26 (237.7,270.83)	229.39 (214.8,243.98)
	2028	3728.29 (2403.57,5053.01)	274823.5 (261281.23,288365.78)	3607.08 (3339.28,3874.89)	241.12 (222.58,259.66)	252.59 (232.45,272.73)	227.83 (210.14,245.51)
	2029	3703.18 (2116.15,5290.22)	1126436.22 (1062685.87,1190186.56)	3594.54 (3275.98,3913.11)	239.57 (217.56,261.58)	250.98 (227.08,274.89)	226.34 (205.39,247.29)
	2030	3678.38 (1816.33,5540.43)	5169392.82 (4842366.26,5496419.38)	3582.33 (3210.42,3954.24)	238.09 (212.46,263.73)	249.43 (221.58,277.27)	224.93 (200.56,249.3)
	2031	3654.08 (1505.33,5802.83)	25344018.86 (23560686.12,27127351.6)	3569.94 (3142.24,3997.63)	236.61 (207.2,266.02)	247.87 (215.92,279.82)	223.51 (195.59,251.43)
	2032	3629.83 (1183.83,6075.83)	129240868.43 (119061823.8,139419913)	3557.31 (3071.45,4043.17)	235.13 (201.8,268.46)	246.32 (210.12,282.53)	222.07 (190.46,253.69)
	2033	3605.99 (852.85,6359.13)	674890813.69 (614848405.8,734933221.6)	3545.08 (2998.83,4091.33)	233.68 (196.3,271.06)	244.81 (204.21,285.42)	220.68 (185.26,256.1)
	2034	3582.39 (513.12,6651.66)	3573354470.29 (3211639337,3935069604)	3533.34 (2924.64,4142.04)	232.29 (190.74,273.84)	243.36 (198.22,288.49)	219.36 (180.01,258.71)
	2035	3558.88	19055951174.69	3521.82	230.96	241.95	218.11

Conditions	Years	ASPR(95% UI, per 100,000)			ASYR(95% UI, per 100,000)		
		Both sexes	Females	Males	Both sexes	Females	Males
		(165.32,6952.44)	(16853366930,21258535420)	(2848.77,4194.88)	(185.13,276.8)	(192.16,291.74)	(174.72,261.5)
	2036	3536.18<br>(-189.92,7262.28)	101896520729.51<br>(88450054095,115343000000)	3510.39<br>(2771.13,4249.65)	229.65<br>(179.42,279.88)	240.56<br>(186.01,295.11)	216.86<br>(169.34,264.38)

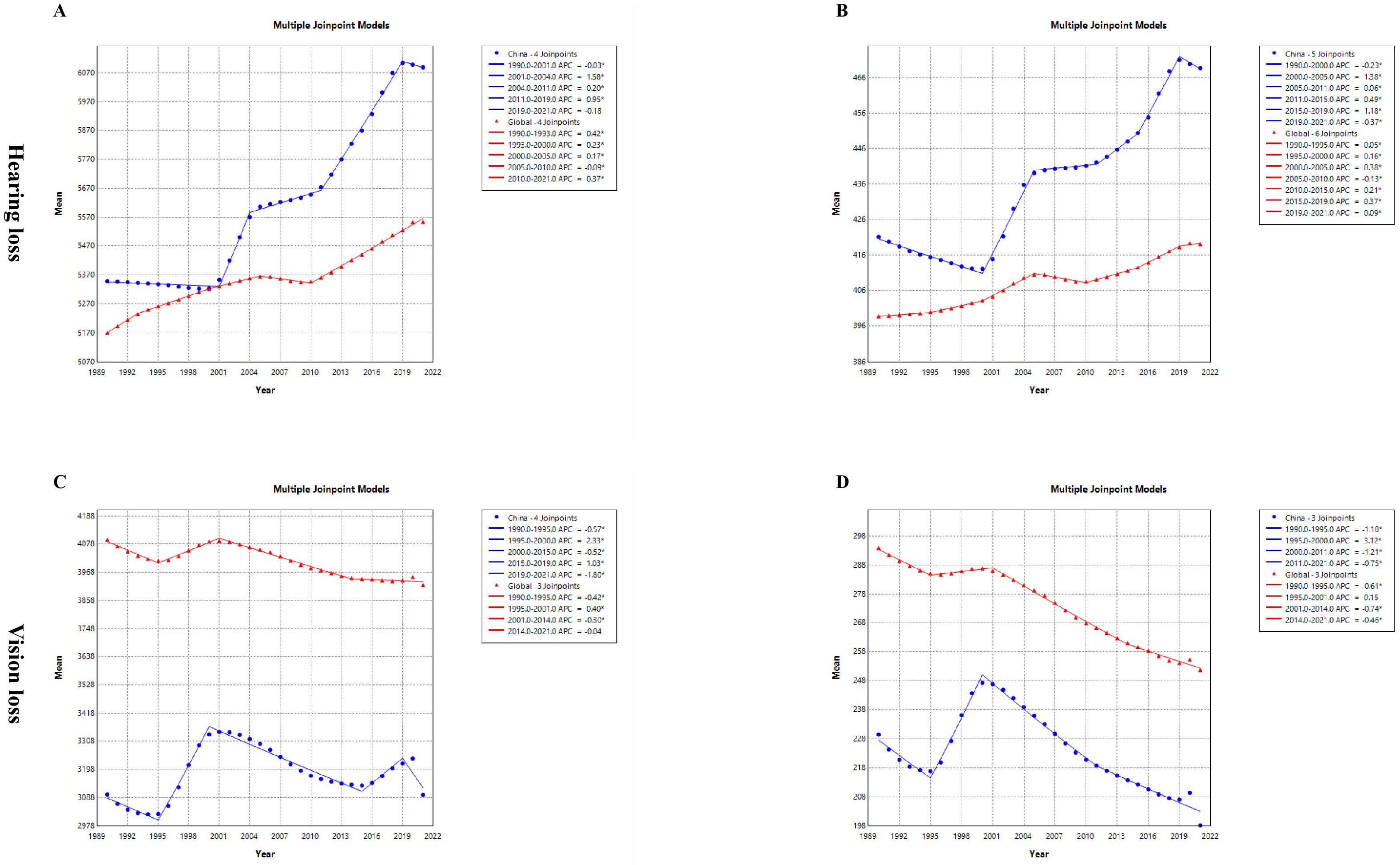

Figure S1 Annual Percentage Change in age-standardized rehabilitation needs for hearing loss and vision loss in China and globally from 1990 to 2021. **A** ASPR for hearing loss requiring rehabilitation in China; **B** ASYR for hearing loss requiring rehabilitation globally; **C** ASPR for vision loss requiring rehabilitation in China; **D** ASYR for vision loss requiring rehabilitation globally. ASPR: age-standardized prevalence rate; ASYR: age-standardized YLDs rate. (* indicates p-value < 0.05; it indicates that the Annual Percent Change (APC) is significantly different from zero at the alpha = 0.05 level.) Final selected model: 6 Joinpoints.

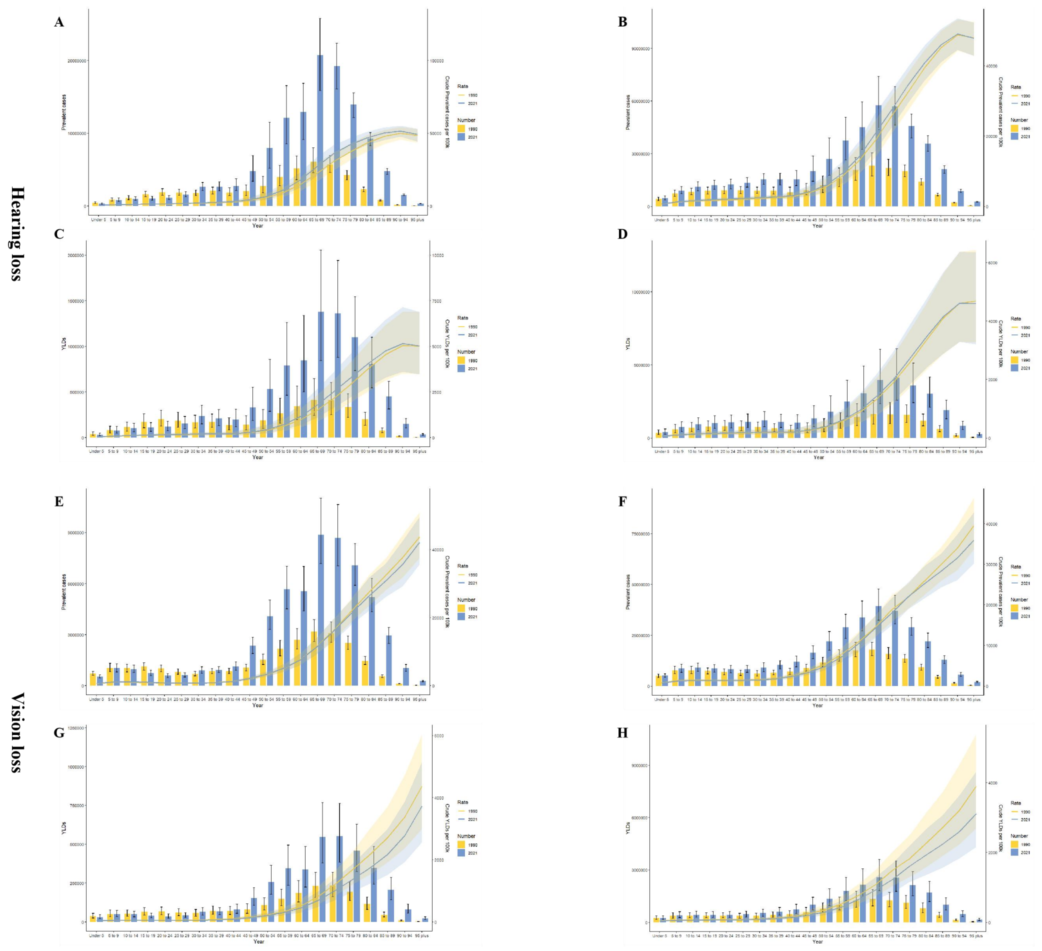

Figure S2 Comparison of the prevalence and YLDs, along with their crude rates, for hearing loss and vision loss rehabilitation needs by age group in China and globally from 1990 to 2021. **A** Prevalent cases and CPR for hearing loss requiring rehabilitation in China; **B** Prevalent cases and CPR for hearing loss requiring rehabilitation globally; **C** YLDs counts and CYR for hearing loss requiring rehabilitation in China; **D** YLDs counts and CYR for hearing loss requiring rehabilitation globally; **E** Prevalent cases and CPR for vision loss requiring rehabilitation in China; **F** Prevalent cases and CPR for vision loss requiring rehabilitation globally; **G** YLDs counts and CYR for vision loss requiring rehabilitation in China; **H** YLDs counts and CYR for vision loss requiring rehabilitation globally. CPR: crude prevalence rate; CYR: crude YLDs rate; Bar charts represent counts; lines represent crude rates.

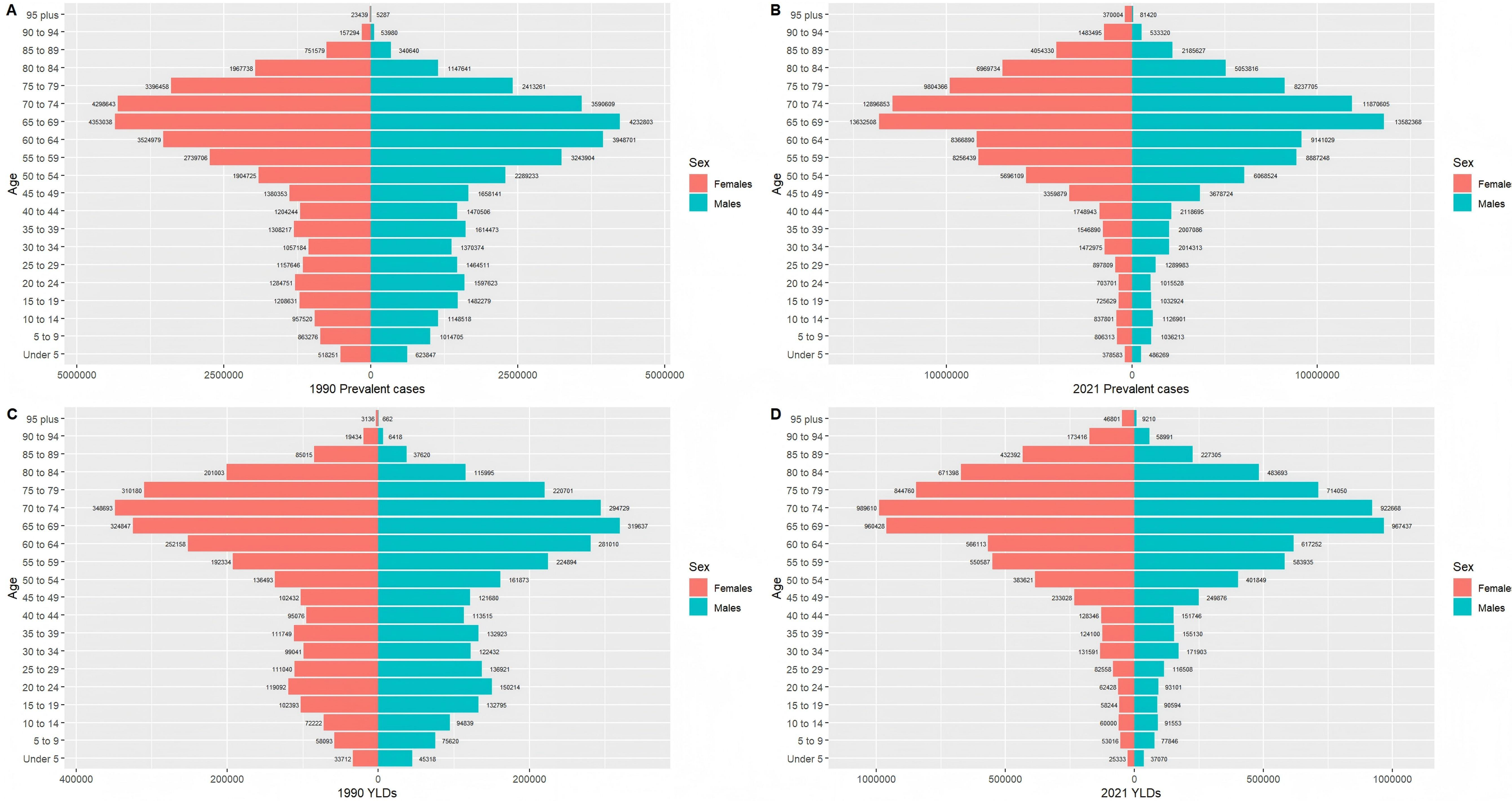

Figure S3 Rehabilitation needs for sensory impairments: Prevalence and YLDs among males and females across different age groups in China, 1990 and 2021. A Prevalent cases in 1990; B Prevalent cases in 2021; C YLDs in 1990; D YLDs in 2021.

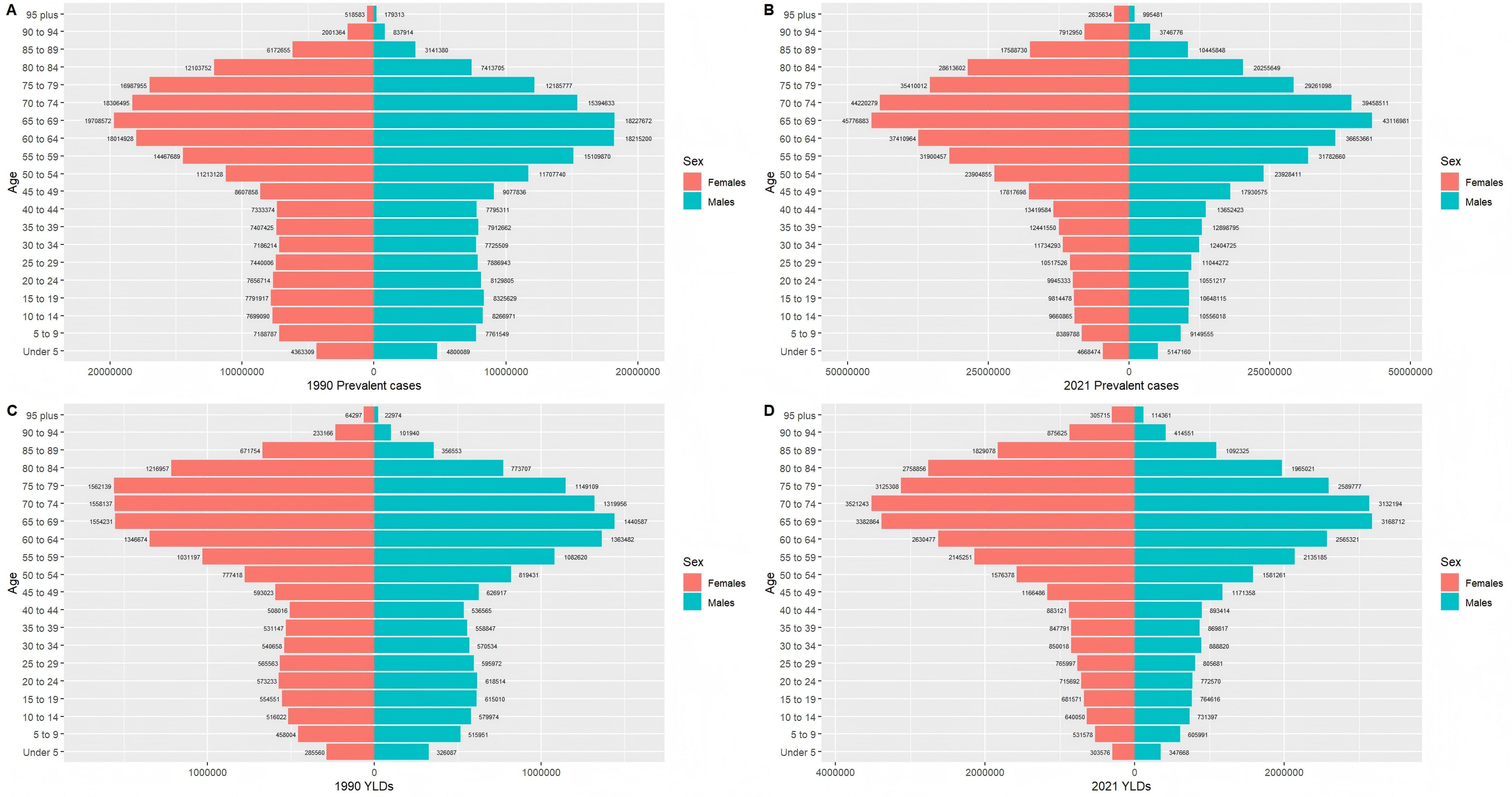

Figure S4 Rehabilitation needs for sensory impairments: Prevalence and YLDs among males and females across different age groups globally, 1990 and 2021. A Prevalent cases in 1990; B Prevalent cases in 2021; C YLDs in 1990; D YLDs in 2021.

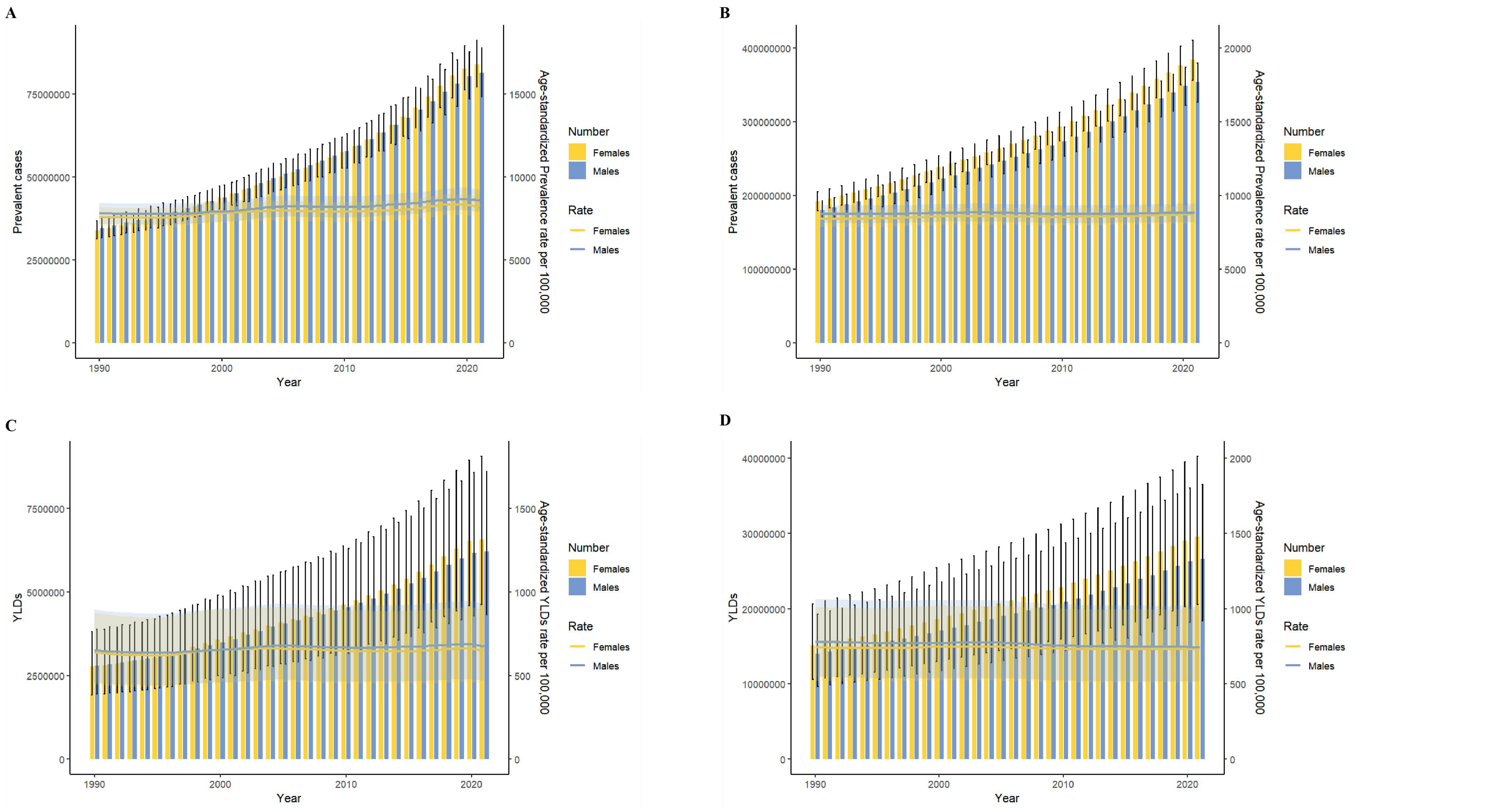

Figure S5 Comparison of full-age cases and age-standardized rates of prevalence and YLDs for sensory impairments rehabilitation needs among males and females in China and globally from 1990 to 2021. A Prevalent cases and ASPR in China; B Prevalent cases and ASPR globally; C YLDs counts and ASYR in China. D YLDs counts and ASYR globally. ASPR: age-standardized prevalence rate; ASYR: age-standardized YLDs rate; Bar charts represent counts; lines represent age-standardized rates.

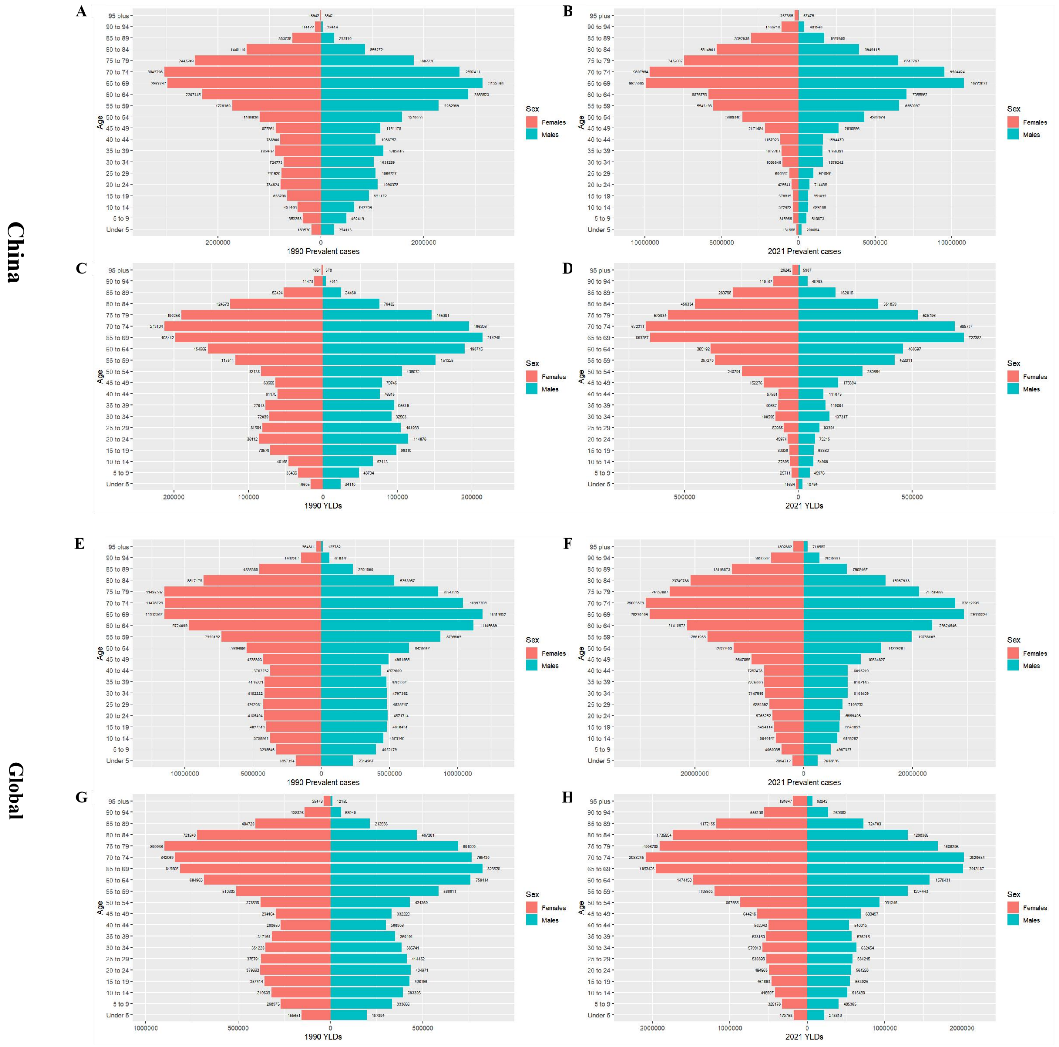

Figure S6 Rehabilitation needs for hearing loss: Prevalence and YLDs among males and females across different age groups in China and globally, 1990 and 2021. **A** Prevalent cases in 1990 in China; **B** Prevalent cases in 2021 in China; **C** YLDs in 1990 in China; **D** YLDs in 2021 in China; **E** Prevalent cases in 1990 globally; **F** Prevalent cases in 2021 globally; **G** YLDs in 1990 globally; **H** YLDs in 2021 globally.

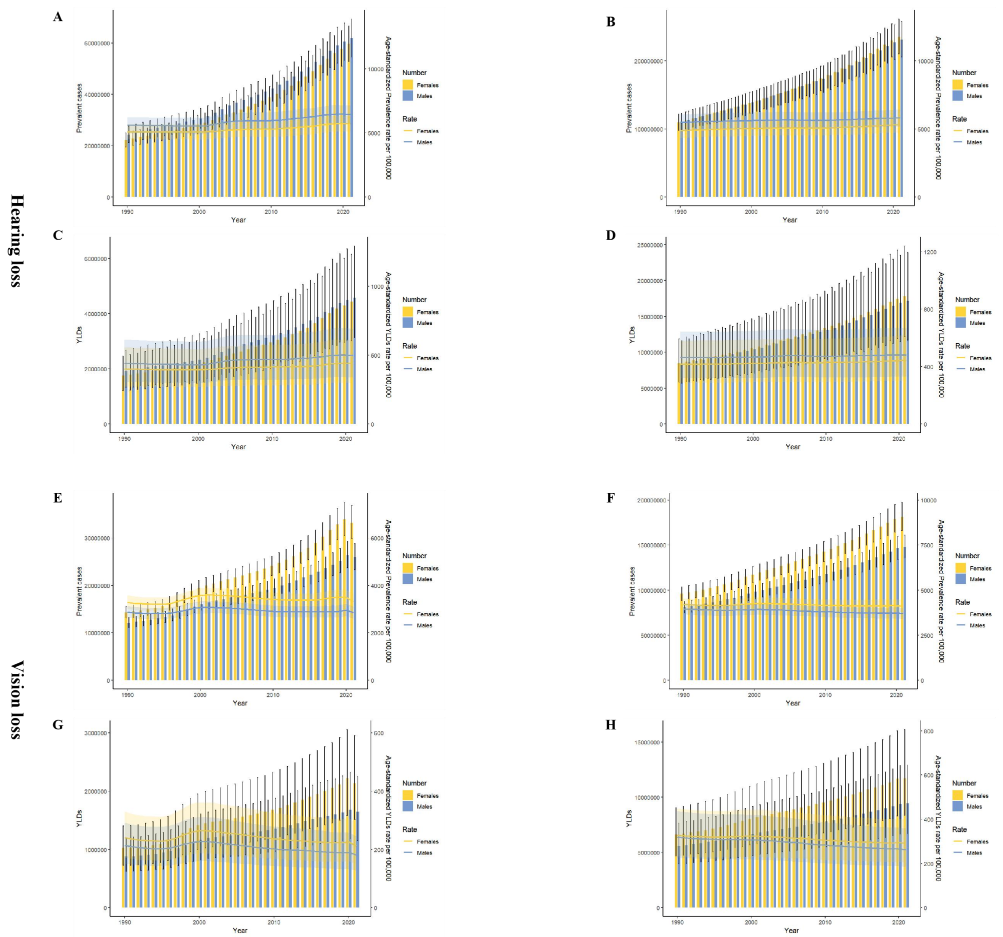

Figure S7 Comparison of full-age cases and age-standardized rates of prevalence and YLDs for hearing loss and vision loss rehabilitation needs among males and females in China and globally from 1990 to 2021. **A** Prevalent cases and ASPR for hearing loss requiring rehabilitation in China; **B** Prevalent cases and ASPR for hearing loss requiring rehabilitation globally; **C** YLDs counts and ASYR for hearing loss requiring rehabilitation in China. **D** YLDs counts and ASYR for hearing loss requiring rehabilitation globally; **E** Prevalent cases and ASPR for vision loss requiring rehabilitation in China; **F** Prevalent cases and ASPR for vision loss requiring rehabilitation globally; **G** YLDs counts and ASYR for vision loss requiring rehabilitation in China. **H** YLDs counts and ASYR for vision loss requiring rehabilitation globally. ASPR: age-standardized prevalence rate; ASYR: age-standardized YLDs rate; Bar charts represent counts; lines represent age-standardized rates.

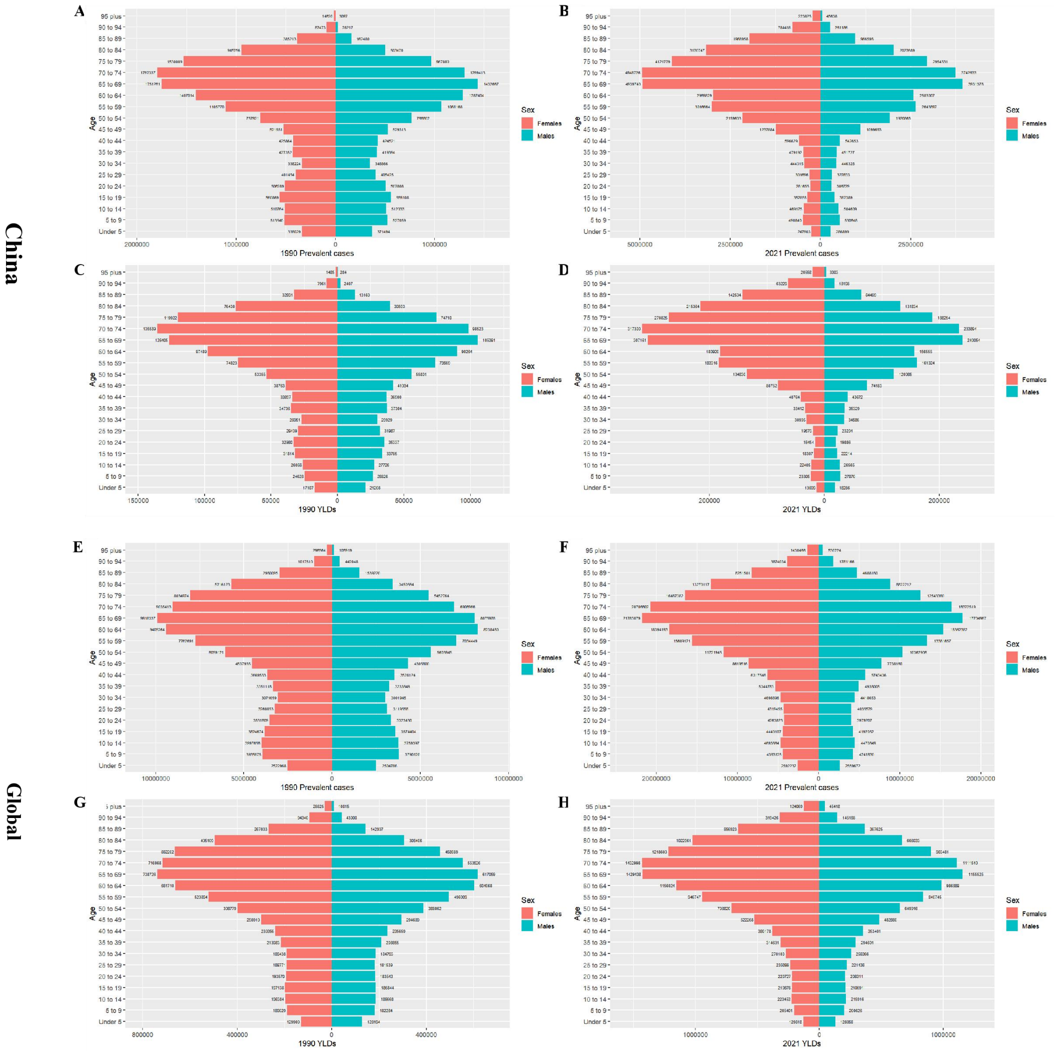

Figure S8 Rehabilitation needs for vision loss: Prevalence and YLDs among males and females across different age groups in China and globally, 1990 and 2021. **A** Prevalent cases in 1990 in China; **B** Prevalent cases in 2021 in China; **C** YLDs in 1990 in China; **D** YLDs in 2021 in China; **E** Prevalent cases in 1990 globally; **F** Prevalent cases in 2021 globally; **G** YLDs in 1990 globally; **H** YLDs in 2021 globally.

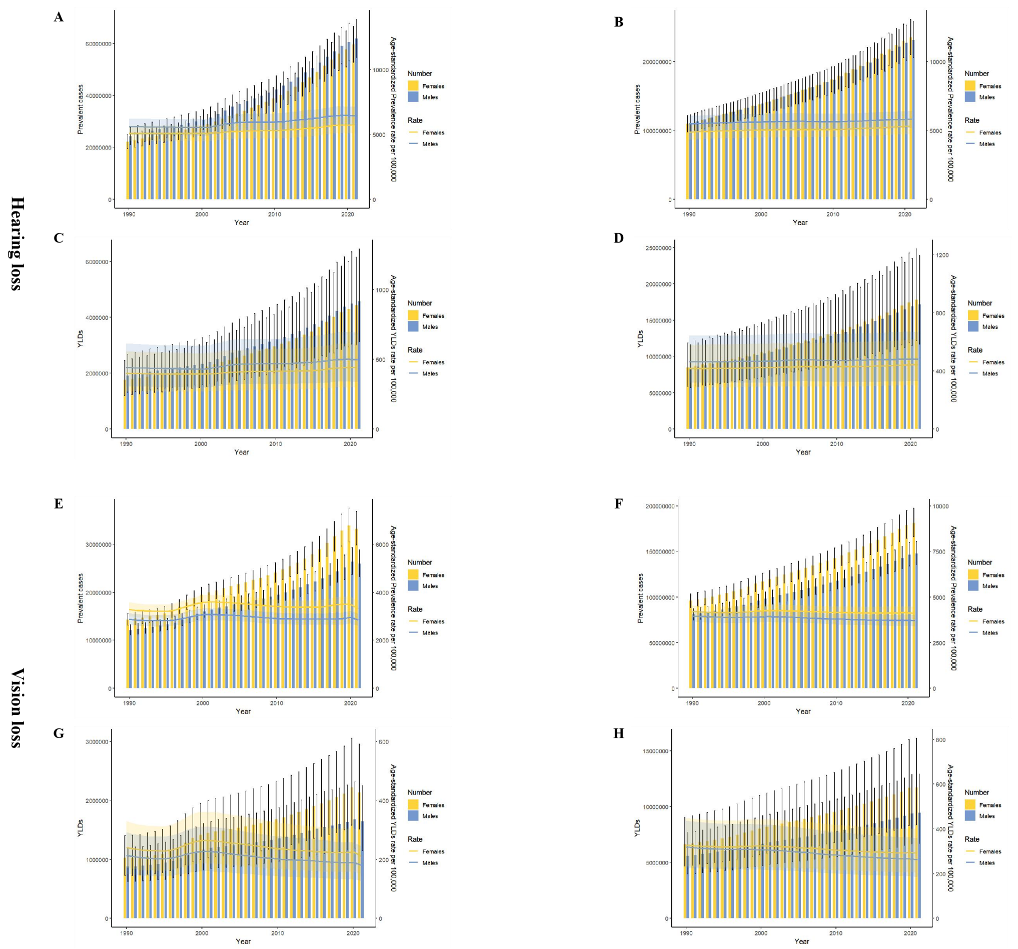

Figure S9 Comparison of full-age cases and age-standardized rates of prevalence and YLDs for hearing loss and vision loss rehabilitation needs among males and females in China and globally from 1990 to 2021. **A** Prevalent cases and ASPR for hearing loss requiring rehabilitation in China; **B** Prevalent cases and ASPR for hearing loss requiring rehabilitation globally; **C** YLDs counts and ASYR for hearing loss requiring rehabilitation in China. **D** YLDs counts and ASYR for hearing loss requiring rehabilitation globally; **E** Prevalent cases and ASPR for vision loss requiring rehabilitation in China; **F** Prevalent cases and ASPR for vision loss requiring rehabilitation globally; **G** YLDs counts and ASYR for vision loss requiring rehabilitation in China. **H** YLDs counts and ASYR for vision loss requiring rehabilitation globally. ASPR: age-standardized prevalence rate; ASYR: age-standardized YLDs rate; Bar charts represent counts; lines represent age-standardized rates.

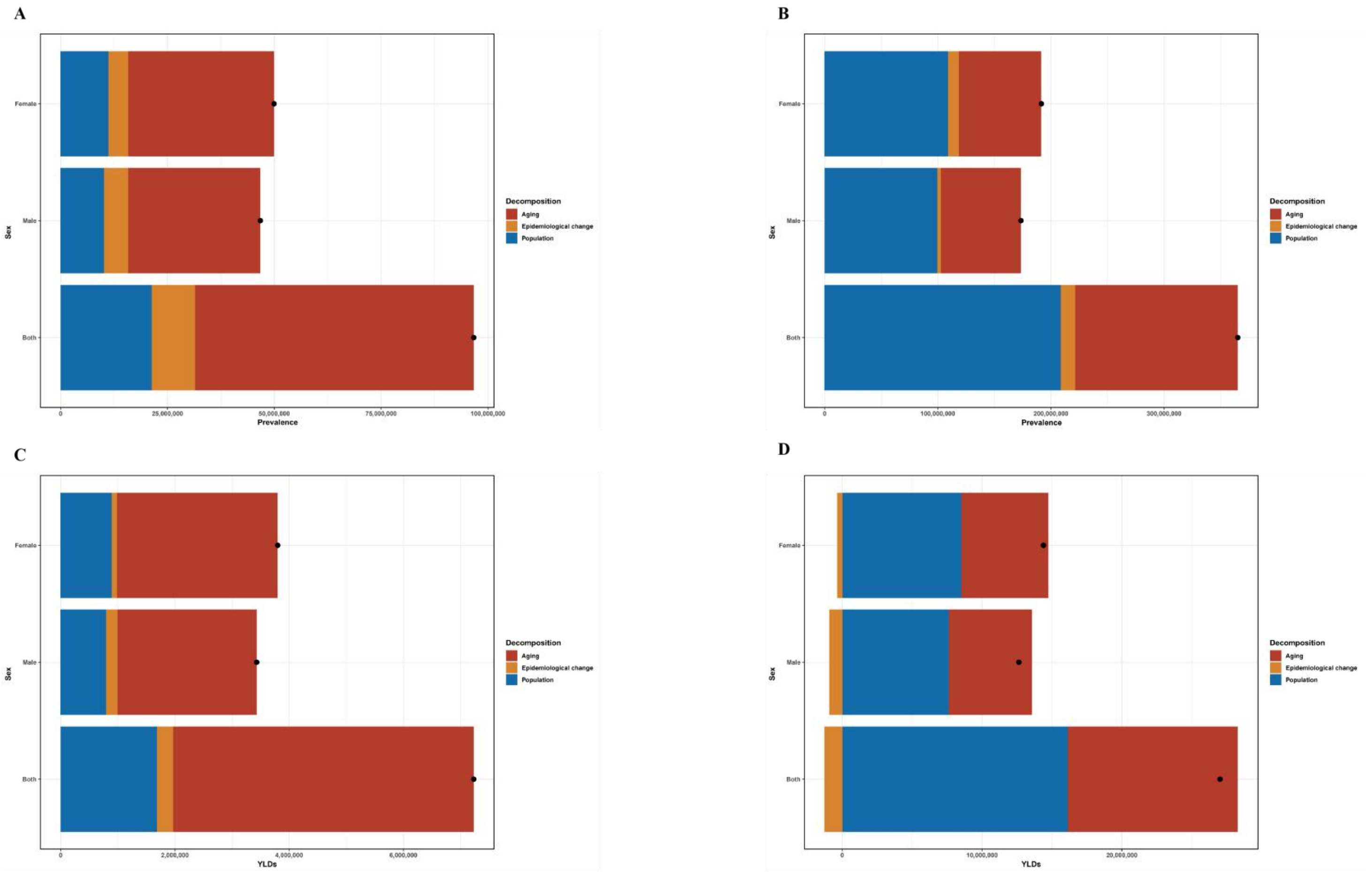

Figure S10 A decomposition analysis, which shows the contribution of changes in population aging, population growth, and epidemiological changes to changes in sensory impairments rehabilitation needs from 1990 to 2021 in China and globally. A Prevalence in China; B Prevalence globally; C YLDs in China; D YLDs globally. Brown represents the percentage contribution of population aging, yellow represents epidemiological changes and blue represents population growth. The first row contains data for females, the second row for males, and the third row for all genders.

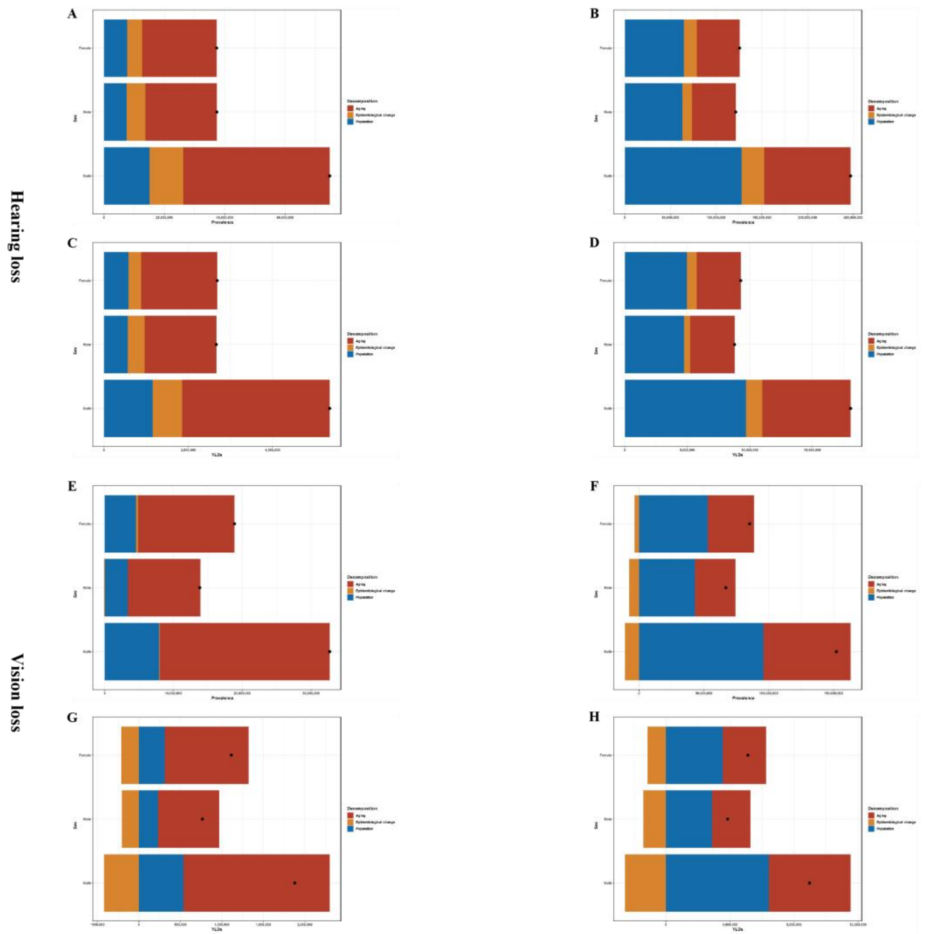

Figure S11 A decomposition analysis, which shows the contribution of changes in population aging, population growth, and epidemiological changes to changes in hearing loss and vision loss rehabilitation needs from 1990 to 2021 in China and globally. **A** Prevalence for hearing loss requiring rehabilitation in China; **B** Prevalence for hearing loss requiring rehabilitation globally; **C** YLDs for hearing loss requiring rehabilitation in China; **D** YLDs for hearing loss requiring rehabilitation globally; **E** Prevalence for vision loss requiring rehabilitation in China; **F** Prevalence for vision loss requiring rehabilitation globally; **G** YLDs for vision loss requiring rehabilitation in China; **H** YLDs for vision loss requiring rehabilitation globally. Brown represents the percentage contribution of population aging, yellow represents epidemiological changes and blue represents population growth. The first row contains data for females, the second row for males, and the third row for all genders.

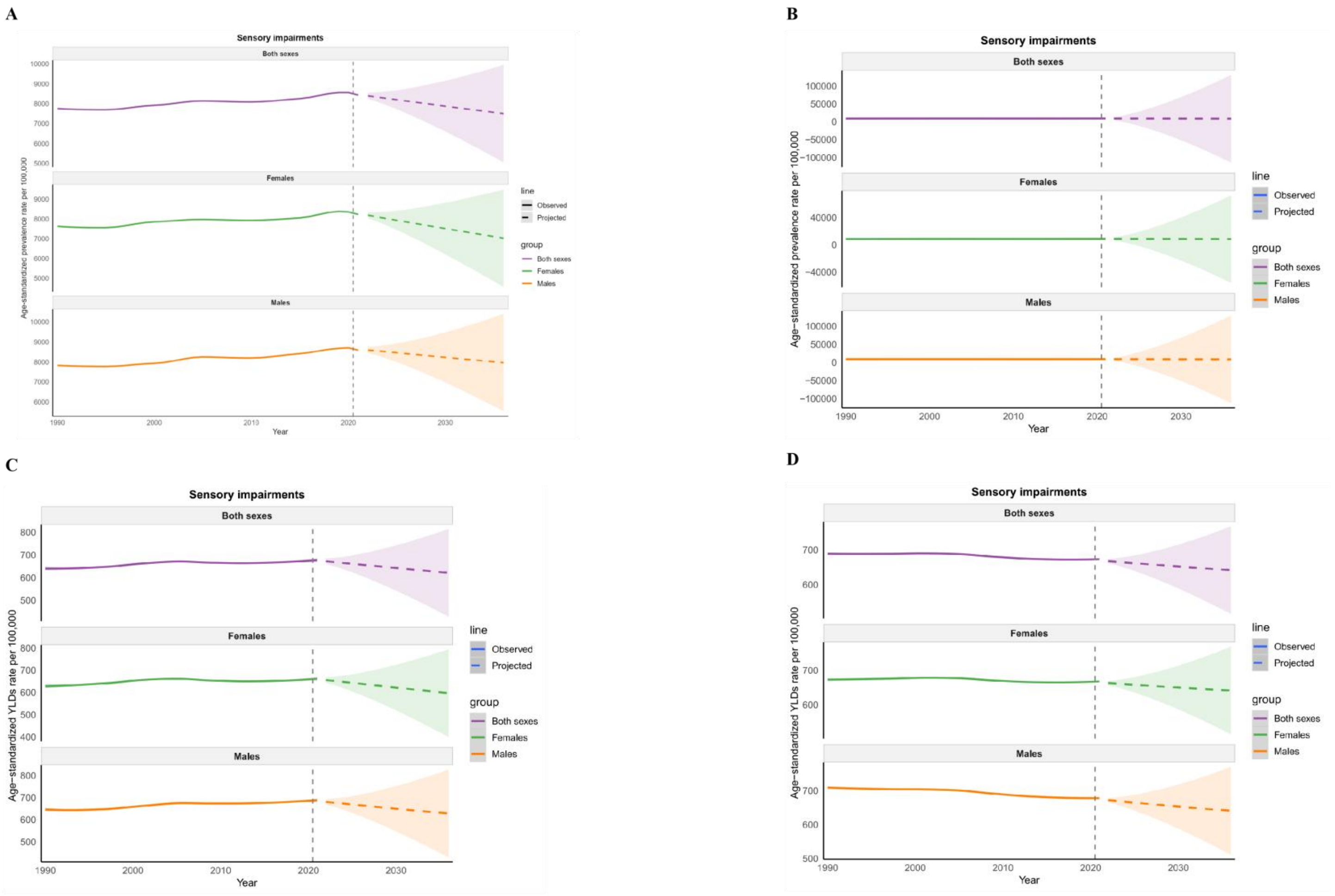

Figure S12 Temporal trends in the ASPR and ASYR for sensory impairments rehabilitation needs from 1990 to 2036 for both males and females in China and globally. Solid lines represent observed ASRs, and dashed lines represent ASRs predicted by the BAPC model. A ASPR in China; B ASPR globally; C ASYR in China; D ASYR globally. ASPR: age-standardized prevalence rate; ASYR: age-standardized YLDs rate; BAPC: Bayesian age-period-cohort.

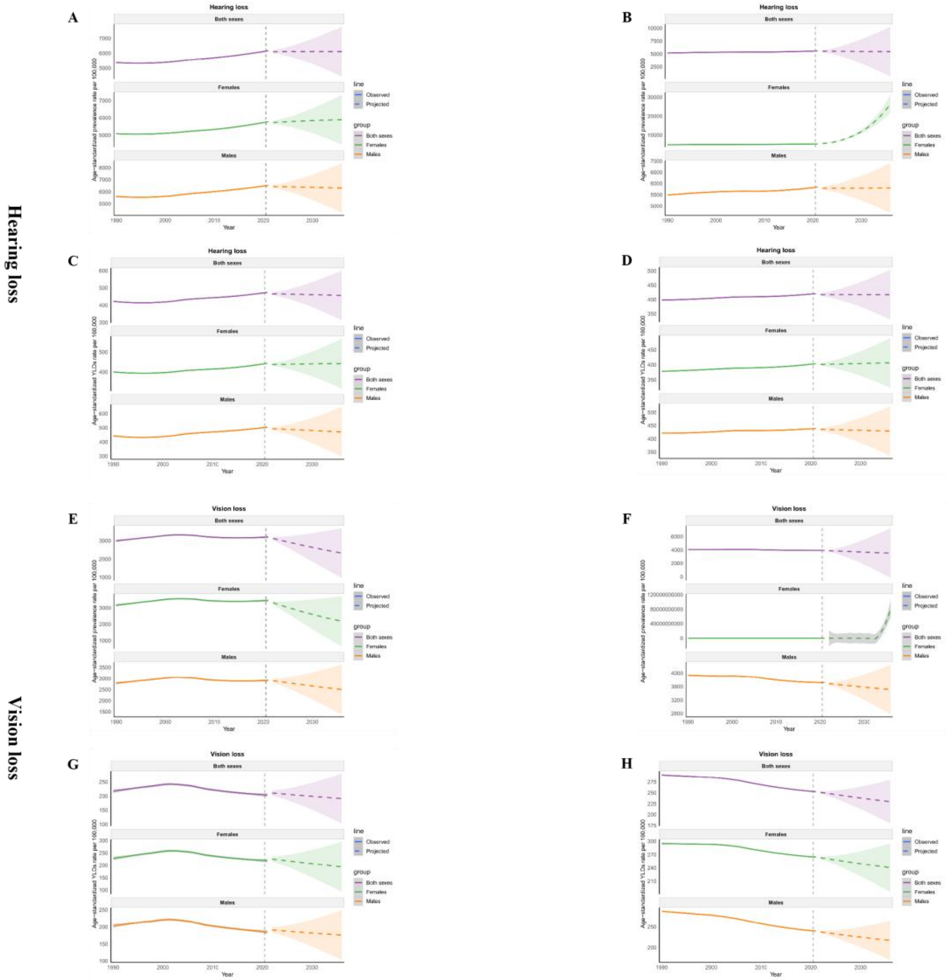

Figure S13 Temporal trends in the ASPR and ASYR for hearing loss and vision loss rehabilitation needs from 1990 to 2036 for both males and females in China and globally. Solid lines represent observed ASRs, and dashed lines represent ASRs predicted by the BAPC model. **A** ASPR for hearing loss requiring rehabilitation in China; **B** ASPR for hearing loss requiring rehabilitation globally; **C** ASYR for hearing loss requiring rehabilitation in China; **D** ASYR for hearing loss requiring rehabilitation globally; **E** ASPR for vision loss requiring rehabilitation in China; **F** ASPR for vision loss requiring rehabilitation globally; **G** ASYR for vision loss requiring rehabilitation in China; **H** ASYR for vision loss requiring rehabilitation globally. ASPR: age-standardized prevalence rate; ASYR: age-standardized YLDs rate; BAPC: Bayesian age-period-cohort.
